# Whole-Genome Resequencing-Based Selection-Signal and Association Analyses Prioritize Candidate Genes and Haplotypes for PRRS Resistance-Related Traits in Pigs

**DOI:** 10.3390/ani16142218

**Published:** 2026-07-17

**Authors:** Meng-Jie Lian, Jia-Qi Wang, Ai-Shi Xu, Zhi Cao, Shi-Ying Zhou, Hong-Ming Yuan, Zi-Cong Xie, Hong-Sheng Ouyang, Da-Xin Pang, Dong-Mei Lv

**Affiliations:** College of Animal Sciences, Jilin University, Changchun 130062, China; lianmj25@163.com (M.-J.L.); wangjqi95@163.com (J.-Q.W.); xuaishi2008@aliyun.com (A.-S.X.); caozhi25@mails.jlu.edu.cn (Z.C.); shiyingz24@mails.jlu.edu.cn (S.-Y.Z.); yuanhongming@jlu.edu.cn (H.-M.Y.); xzc@jlu.edu.cn (Z.-C.X.); ouyh@jlu.edu.cn (H.-S.O.)

**Keywords:** porcine reproductive and respiratory syndrome, PRRS resistance, whole-genome resequencing, genome-wide association study, selection-signal analysis, candidate genes, haplotype analysis

## Abstract

Porcine reproductive and respiratory syndrome (PRRS), caused by PRRSV, causes substantial economic losses in the swine industry. Because viral variability and host genetic complexity limit conventional control, identifying host genetic factors associated with PRRS resistance through genomic approaches is important for disease-resistant breeding. In this study, 699 pigs were immunized with a PRRSV vaccine, 135 were selected for PRRSV infection experiments, and 133 were retained for whole-genome resequencing after two-stage phenotypic screening based on post-immunization and post-infection profiles. Genome-wide selection-signal analysis identified 12 highly differentiated regions (F_st_ > 0.15), annotated to 11 candidate genes: *NFXL1*, *NIPAL1*, *CHIC2*, *LOC100623351*, *LOC100513671*, *LOC100513484*, *CENPC*, *STAP1*, *UBA6*, *GNRHR*, and *LOC100512727*. The original exploratory GWAS identified candidate association signals, including signals annotated to *PYGM*, *NFXL1*, *KIAA1324L*, and *FLNC*; after PC1/PC2 adjustment, *NFXL1* retained exploratory support, and additional exploratory signals were observed. Public PRRSV-related transcriptomic datasets provided additional expression-level evidence, with *NIPAL1* and *PYGM* showing increased expression in PRRSV-infected porcine alveolar macrophages. Functional enrichment and variant-level analyses supported the biological relevance of the prioritized candidate gene set, particularly the chromosome 8 *NFXL1* region, where the A-C-G haplotype was more frequent in resistant pigs. These findings provide useful genetic clues for further validation and PRRS resistance breeding.

## Simple Summary

Porcine reproductive and respiratory syndrome is a serious infectious disease that causes reproductive problems, breathing illness, and major economic losses in pig production. Vaccines and farm management help control the disease, but they do not always provide enough protection because the virus changes easily. This study aimed to find inherited genetic features that may help identify pigs with better natural resistance to this disease. Pigs were first screened after vaccination and then after exposure to the virus, and animals with clearly different disease-response patterns were selected for detailed genetic analysis. By comparing genetic differences between resistant and susceptible pigs and combining these results with public data showing how genes behave during infection, this study identified several candidate genes and genetic regions related to disease response. A region on chromosome 8, especially around genes named *NFXL1* and *NIPAL1*, was highlighted, and a specific genetic pattern in this region was more common in resistant pigs. These findings provide useful clues for future testing and may support breeding programs aimed at improving disease resistance in pigs.

## 1. Introduction

Pigs are among the most important livestock species worldwide, and commercial breeds such as Large White, Landrace, and Duroc are widely used in pig production because of their growth performance, feed efficiency, and meat quality [1]. However, infectious diseases remain a major constraint on the sustainable development of the swine industry. Porcine reproductive and respiratory syndrome (PRRS) is one of the most economically important infectious diseases affecting pigs globally. It is characterized by reproductive disorders in pregnant sows, respiratory disease in piglets and growing pigs, and immunosuppression. These clinical consequences have important animal-welfare implications, including abortion, stillbirth or weak-born piglets, respiratory distress, increased susceptibility to secondary infections, and mortality [2]. In addition to welfare concerns, PRRS causes substantial economic losses through reproductive failure, reduced growth performance, increased mortality, treatment costs, and production instability. Previous economic analyses have reported major losses in the swine industry in both the United States and China [3,4].

PRRS is caused by porcine reproductive and respiratory syndrome virus (PRRSV), an RNA virus characterized by high genetic variability, antigenic drift, immune evasion, and frequent immunosuppressive effects. Although vaccination and biosecurity measures provide partial protection, the continuous mutation and emergence of new viral strains limit the long-term effectiveness of conventional prevention and control strategies. Therefore, improving host genetic resistance has become an important complementary strategy for PRRSV control. In this context, identifying SNPs and candidate genes associated with PRRS resistance is essential for understanding host resistance mechanisms and supporting disease-resistant breeding.

Genomic selection-signal analysis and genome-wide association studies (GWAS) provide useful approaches for identifying genomic regions and variants associated with complex disease-response traits. PRRS host genetics has been extensively investigated, and several PRRSV-response loci and genetic targets have been reported. The most well-characterized naturally segregating locus is the SSC4 region tagged by the WUR10000125 SNP, which is located near the *GBP* gene cluster and has been associated with viral load and weight gain after experimental PRRSV infection [5]. Within this region, a splice-site variant in *GBP5* has been proposed as a candidate quantitative trait nucleotide for the major SSC4 QTL [6]. Additional GWAS and QTL studies have also reported PRRSV-response-related loci on other chromosomes, including SSC7 and SSC17, using traits such as viral load, weight gain, reproductive response, fetal survival, or susceptibility-related phenotypes [7,8]. In addition to naturally segregating genetic variation, CD163-based gene-editing studies have demonstrated that targeting a PRRSV entry receptor can produce pigs or cells with strong resistance to PRRSV infection [9,10].

Despite these advances, several limitations remain. Many previous studies focused on viral load, growth performance, reproductive loss, fetal survival, or gene-expression traits, whereas fewer studies have combined post-immunization antibody response with post-infection PRRSV genome detection to define divergent host-response phenotypes. In addition, reported loci may vary across pig populations, PRRSV strains, infection models, and phenotype definitions, and most association signals still require independent validation and functional interpretation. Therefore, complementary phenotype-guided genomic studies, together with external transcriptomic evidence and variant-level analyses, may help identify additional candidate loci involved in PRRSV-related host response.

Building on this rationale, in this study, Large White, Duroc, and Landrace pigs from a commercial farm were used as the study population after PRRSV immunization. The pigs were first screened based on ELISA-measured antibody responses. Selected pigs were then subjected to PRRSV infection experiments, followed by post-infection PRRSV genome detection and antibody testing. Based on this two-stage phenotypic screening strategy, phenotypically divergent resistant and susceptible groups were established for whole-genome resequencing and downstream genetic analyses. This study aimed to identify PRRS resistance-related candidate regions, SNPs, and genes by integrating genome-wide selection-signal analysis and GWAS. To further interpret the prioritized candidates, public PRRSV-related transcriptomic datasets were used to assess candidate-gene expression patterns, and functional enrichment, allele-frequency comparison, linkage disequilibrium, and haplotype analyses were performed to characterize the biological and variant-level context of the candidate regions.

## 2. Materials and Methods

### 2.1. Animal Population, PRRSV Vaccination, Experimental Infection, and Two-Stage Phenotypic Screening

The initial cohort comprised 699 privately owned pigs from a commercial farm population that met the requirements for PRRSV vaccination, blood sampling, and subsequent phenotypic screening. The pigs were initially included based on study eligibility and sample availability rather than clinical signs observed after infection. The cohort included 629 females and 70 males, with ages ranging from 8 to 200 weeks, and covered breeding pigs, replacement gilts, and grower/finisher pigs at different productive stages. Detailed individual metadata, including sex, age in weeks, pig type, current parity, productive stage, breeding status, current status, post-immunization PRRSV antibody S/P ratio, and PRRSV antibody result, are provided in Supplementary Table S1.

A total of 699 pigs were immunized with a commercial modified-live PRRSV vaccine, Ingelvac^®^ PRRS MLV (Boehringer Ingelheim, Ingelheim, Germany), based on the VR-2332 strain. Each pig received one dose, equivalent to 2 mL, by intramuscular injection, and each dose contained at least 10^4.8^ TCID_50_ of PRRSV [11]. After immunization, serum anti-PRRSV antibodies were detected using a porcine reproductive and respiratory syndrome virus ELISA antibody detection kit (Jinnuo Diagnostics, Beijing, China). Based on the serum S/P values, the pigs falling within the predefined S/P ranges were classified into low, medium, and high antibody-response groups, with S/P values of ≤0.9, 1.3–1.8, and ≥2.0, respectively. These thresholds were used as phenotypic stratification criteria rather than diagnostic cutoffs. Because the subsequent PRRSV infection experiment and whole-genome resequencing could not be performed for all 699 pigs, S/P-based stratification was used to select pigs covering weak, medium, and strong post-immunization antibody-response ranges. The intervals between adjacent groups were retained to avoid borderline S/P values and reduce potential misclassification. Post-immunization antibody responses were used as the first phenotypic screening layer to characterize individual variation in vaccine-induced immune response.

To further evaluate post-infection responses, 135 pigs with different post-immunization antibody-response levels were selected for PRRSV challenge. PRRSV-positive serum containing the highly pathogenic PRRSV strain JXA1 was used as the infection inoculum. The inoculum was administered by nasal spray to mimic respiratory exposure and by intramuscular injection at a dose of 1500 TCID_50_ per pig. After the PRRSV infection experiment, clinical observations were made during routine farm monitoring. Available individual-level reproductive records were reviewed to summarize abortion in sows where recorded. Respiratory signs such as cough were not systematically scored at the individual level under group-housed commercial-farm conditions. These clinical observations were used only as supportive post-infection information and were not directly used as quantitative criteria for subsequent grouping analyses. At 15 days post-infection, whole blood and throat swabs were collected from the 135 pigs for PRRSV genome detection by RT-PCR, and serum samples were collected for post-infection antibody detection by ELISA.

According to the combined post-immunization antibody responses, post-infection PRRSV genome detection results, and post-infection antibody profiles, resistant and susceptible groups were constructed using this dual phenotypic screening strategy. A total of 133 pigs were finally retained for whole-genome resequencing.

### 2.2. DNA Extraction, Whole-Genome Resequencing, and Variant Calling

Venous blood samples (4 mL per pig) were collected, and DNA was extracted from blood clot samples. DNA samples that met the quality-control criteria were sent to Beijing Novogene Technology Co., Ltd. (Beijing, China) for sequencing in paired-end 150-bp (PE150) mode on the DNBSEQ-T7 platform (MGI, Shenzhen, China). The sequencing depth was 10×, and approximately 30 GB of sequencing data were generated for each individual. Clean reads were obtained by filtering out paired reads containing adapters, reads in which unidentified nucleotides (N) accounted for ≥10% of either read, and reads in which low-quality bases accounted for ≥50%.

Sscrofa11.1 was used as the reference genome, and clean reads were aligned to the reference genome using BWA (v0.7.17) [12]. The resulting BAM files were sorted and indexed using SAMtools (v1.9) [13]. GATK (v4.3) was used to detect SNPs and generate VCF files. High-quality variant sites with a quality value ≥20, an average depth >4, and a missing rate ≤0.05 were retained. Biallelic SNPs with missing rates ≤20% were further retained using bcftools (v1.19) [14], followed by linkage pruning to generate the final high-quality VCF file.

### 2.3. Population Structure, Linkage Disequilibrium Decay, and Genetic Diversity Analyses

Variant sites located within coding sequence (CDS) regions were extracted from the filtered VCF file based on the reference genome annotation and used for phylogenetic analysis. The extracted variant file was converted into Nexus format using vcf2phylip.py (v2.8), and ModelFinder Plus in IQ-TREE (v1.6.12) was used to select the best-fit evolutionary model for phylogenetic analysis [15]. A phylogenetic tree was then constructed using the maximum likelihood (ML) method to visualize the genetic relationships within the experimental population. Finally, FigTree (v1.4.4) was used for visualization.

Principal component analysis (PCA) was performed using PLINK (v1.9) [16], and the first two principal components were plotted using the ggplot2 package (v3.4.4) in R (4.3.2). Population structure was inferred using ADMIXTURE (v1.3.0) [17]. The assumed number of genetic clusters (K) ranged from 3 to 5, and the --cv=20parameter was used to perform 20 cross-validations for each K value. The optimal ancestry proportion was selected according to the minimum cross-validation (CV) error [17]. LD decay was measured as the squared Pearson correlation coefficient (r^2^) using PopLDdecay (v3.41) [18]. The LD decay distance was defined as the distance at which r^2^ decreased to half of its maximum value.

Runs of homozygosity (ROH) were detected using PLINK [19] with a sliding-window approach. The parameters were set as follows: (1) the minimum ROH length was 500 kb, (2) each ROH contained at least one SNP per 50 kb, (3) at most one heterozygous site was allowed in a sliding window of 50 SNPs, (4) each ROH contained at least 50 consecutive SNPs, (5) the maximum gap between consecutive SNPs within an ROH was ≤100 kb, and (6) the window detection-rate threshold was 5%. The percentage of SNPs located within ROH was calculated to characterize ROH hotspot regions. The integrated data were visualized using the ggplot2 package in R.

According to the LD analysis results, an r^2^ value of 0.25 corresponded to a genetic distance of approximately 50 kb. Therefore, nucleotide diversity (π) was calculated using VCFtools (v4.0) based on 50-kb sliding windows to estimate genetic diversity among groups [20]. The results were plotted using the ggplot2 package in R.

### 2.4. Selection-Signal Detection

VCFtools software (v0.1.16) was used to calculate the population differentiation index (F-statistics, Fst) at the genome-wide level [21], and Fst analysis was performed with a sliding window size of 50 kb. The qqman package in the R language was used to draw the Manhattan diagram, and the genome-wide Fst of the pig population was calculated with reference to the research method of Yang et al. [22] in domestic pigs. The formula is as follows:

$$F_{st}=\frac{MSP\;-\;MSG}{MSP\;+\;\left(n_{c}-\;1\right)MSG}$$

Among them, *MSG* and *MSP* represent the mean square error within the group and the mean square error between the groups, respectively; n_c_ refers to the average sample size between the corrected populations, and n is the number of non-empty groups. According to the calculated *Fst* results, the descending order is sorted, the previous 1% window *Fst* value is used as the indicative threshold, and the Ensembl database is used to perform gene annotation according to the window size. The genome-wide significance threshold line was set to 0.15.

### 2.5. Genome-Wide Association Study

Genome-wide association analysis was performed using PLINK software (v1.9) based on a logistic regression model [23]. The original exploratory GWAS was conducted without PC covariates to identify candidate association signals. In addition, considering the clear breed-related genetic structure observed in the PCA, GWAS was also performed with PC1 and PC2, included as covariates as a complementary population-structure-adjusted analysis. For the original exploratory GWAS, the logistic regression model was defined as follows:

$$log(\frac{p}{1-p})=\beta_{0}+\beta_{1}X$$

For the complementary PC-adjusted GWAS, the model was defined as follows:

$$log(\frac{p}{1-p})=\beta_{0}+\beta_{1}X+\beta_{2}PC1+\beta_{3}\;PC2$$

Here, *p* is the probability of the binary PRRS resistance-related phenotype, X is the genotype encoding of each SNP locus, and PC1 and PC2 are the first two principal components included as covariates in the complementary PC-adjusted GWAS. The model estimated the odds ratio and corresponding P value for each SNP. In the original exploratory GWAS, the original suggestive threshold was defined as P=1/N, and the Bonferroni-corrected threshold was defined as P=0.05/N, where N represents the total number of SNPs tested in the original exploratory GWAS threshold setting. These thresholds were retained for visualization and discovery-stage signal annotation. In the complementary PC-adjusted GWAS, SNPs with $P<1\times 10^{-4}$ were summarized as top-ranked exploratory association signals for comparison with the original GWAS results. In addition, the effective number of independent SNPs after LD pruning $\left(N_{eff}\right)$ was used to calculate a genome-wide suggestive threshold $\left(1/N_{eff}\right)$ for the PC-adjusted analysis [24]. Manhattan and Q-Q plots were generated in R.

### 2.6. Public Transcriptomic Analysis of Candidate Genes

To obtain external expression-level evidence for candidate genes, PRRSV-related public transcriptomic datasets were retrieved from GEO, including GSE174494, GSE89331, GSE296917, GSE277761, GSE304527, GSE84347, GSE75304, and GSE78762 [25,26,27,28,29,30,31,32]. These datasets included relevant porcine immune-cell and tissue sample types, such as porcine alveolar macrophages, pulmonary intravascular macrophages, peripheral blood mononuclear cells, lung dendritic cells, and whole blood.

Candidate-gene expression was extracted using available gene or transcript identifiers. For datasets with published differential-expression tables, reported log_2_ fold changes, *p* values, and adjusted *p* values were used directly. For expression matrices, experimental groups or time points were defined according to the original study design, and expression changes were summarized using the provided normalization or log_2_-transformed group means where appropriate. Two-sided permutation tests were applied only to suitable small sample-level FPKM/RPKM comparisons, whereas raw-count and time-course datasets without formal reanalysis were treated as exploratory trends. The resulting statistical evidence and expression trends were compared with genes prioritized by selection-signal and GWAS analyses to summarize external transcriptomic evidence in PRRSV-related contexts.

### 2.7. Functional Enrichment Analysis

To characterize the potential biological functions of the candidate genes identified by selection-signal analysis and GWAS, genes obtained from the Fst and GWAS analyses were integrated into an Fst and GWAS candidate gene set. Gene Ontology (GO) functional enrichment analysis and Kyoto Encyclopedia of Genes and Genomes (KEGG) pathway enrichment analysis were then performed based on this integrated candidate gene set.

### 2.8. Variant-Level Characterization and Haplotype Analysis

To assess variant-level information within GWAS candidate-gene regions, SNPs located within annotated candidate-gene regions were extracted from the original PLINK genotype dataset according to gene coordinates from the reference genome annotation. For each SNP, allele counts, allele numbers, and allele frequencies were calculated separately in the resistant and susceptible groups. Allele-frequency differences were calculated as resistant minus susceptible, and group differences were assessed using two-sided Fisher’s exact tests followed by BH-FDR correction across the analyzed regional SNPs. Allele-frequency differences were visualized to compare variant-level signals across annotated candidate genes.

For the *NFXL1* candidate region, genotype information for three *NFXL1*-associated candidate SNPs was extracted from the original genotype data, and candidate-allele frequencies were calculated separately in the resistant and susceptible groups. The haplotype-block structure of the *NFXL1* candidate region was visualized as a heatmap using Haploview software (v4.2) [33]. Haplotype frequencies were estimated separately in the resistant and susceptible groups using an expectation–maximization algorithm based on unphased diploid genotypes. Only individuals with complete genotypes at all selected loci were retained for haplotype estimation. Group differences in haplotype frequency distributions were assessed using two-sided Fisher’s exact tests based on estimated haplotype copy counts, followed by multiple-testing correction where applicable.

## 3. Results

### 3.1. Phenotypic Distribution

**Figure 1:**
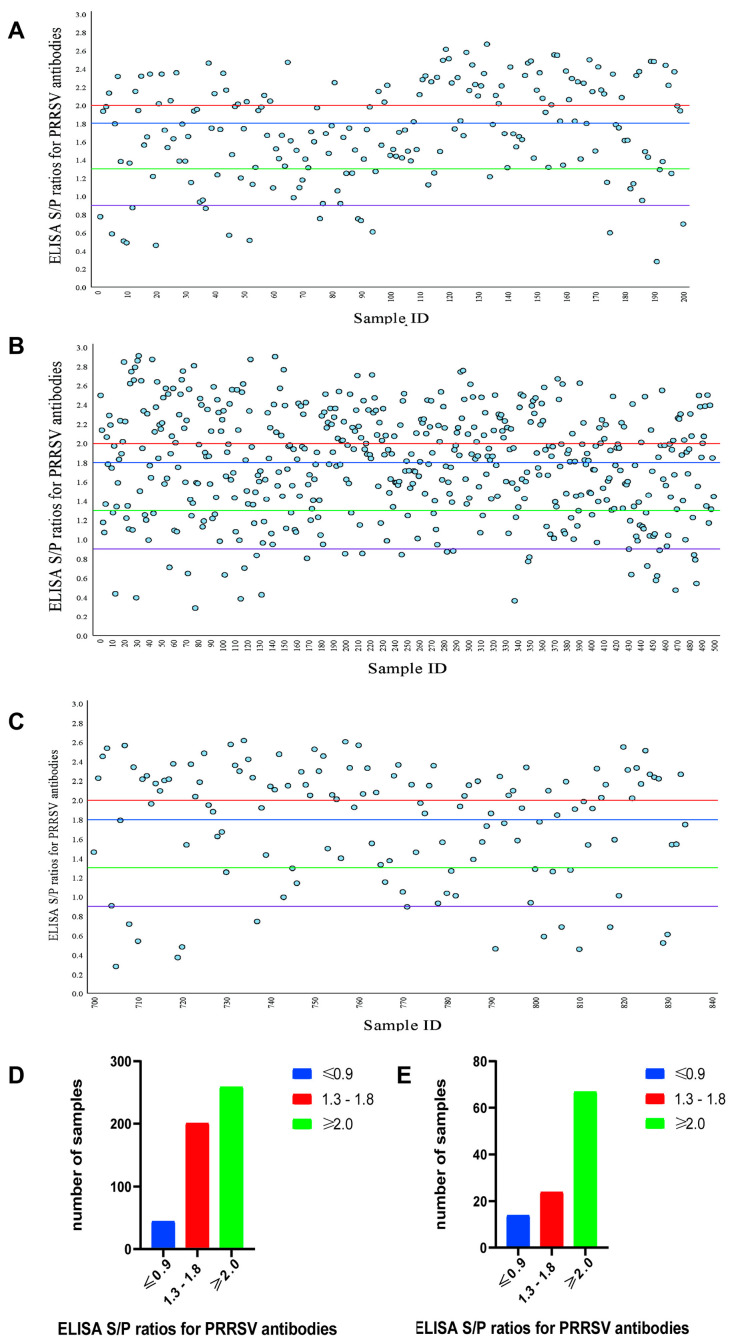
Distribution of antibodies and S/P ratios after PRRS immunization and infection. (**A**,**B**) Distribution of PRRSV antibody S/P value after immunization. (**C**) Distribution of PRRSV antibody S/P value after infection. (**D**) Characteristics of antibody level distribution in 699 pigs after PRRSV vaccination. (**E**) Characterization of antibody level distribution in 135 pigs after PRRSV infection. Each blue dot represents the ELISA S/P ratio of an individual pig. The purple, green, blue, and red horizontal lines indicate S/P values of 0.9, 1.3, 1.8, and 2.0, respectively. These thresholds were used to define the low-antibody-response group (S/P ≤ 0.9), medium-antibody-response group (1.3 ≤ S/P ≤ 1.8), and high-antibody-response group (S/P ≥ 2.0). Pigs with S/P values between 0.9 and 1.3 or between 1.8 and 2.0 were not included in the corresponding antibody-response groups.

Post-immunization ELISA results from 699 pigs showed substantial variation in serum anti-PRRSV antibody responses. According to the predefined S/P thresholds, 45, 201, and 259 pigs fell into the low (S/P ≤ 0.9), medium (S/P 1.3–1.8), and high (S/P ≥ 2.0) antibody-response ranges, respectively, accounting for 6.4%, 28.8%, and 37.1% of the total population (Figure 1A,B,D). This uneven distribution indicated that the three antibody-response strata were not equally represented in the initial population, supporting the use of stratified selection for the subsequent PRRSV infection experiment. Based on the post-immunization antibody distribution, 135 pigs covering different antibody-response ranges were selected for the subsequent PRRSV infection experiment. When categorized according to the predefined S/P thresholds, the selected pigs included 44 pigs with low antibody levels, 44 pigs with medium antibody levels, and 46 pigs with high antibody levels. One additional pig had an S/P value of 1.1499 and was located between the predefined low- and medium-response thresholds.

After experimental infection, whole blood and throat swabs were collected from the 135 pigs for PRRSV genome detection by RT-PCR. The PRRSV-positive rates were 20.74% in throat swabs (28/135) and 6.67% in blood samples (9/135). In total, 32 pigs were identified as PRRSV-positive by either sample type, corresponding to an overall positivity rate of 23.70%, whereas 103 pigs were negative in both sample types (76.30%) (Table 1). Ct values for PRRSV-positive blood and/or throat-swab samples at 15 days post-infection are provided in Supplementary Table S4 as auxiliary indicators of relative PRRSV nucleic acid levels. When summarized by post-immunization antibody-response range, blood PRRSV genome-positive rates were similar among the high, medium, and low antibody-level groups, with 3/46 (6.52%), 3/44 (6.82%), and 3/44 (6.82%) positive pigs, respectively. In throat swabs, the PRRSV genome-positive rates were 8/46 (17.39%) in the high antibody-level group, 9/44 (20.45%) in the medium antibody-level group, and 11/44 (25.00%) in the low antibody-level group. When positivity in either throat swab or blood was considered, the positive rates were 10/46 (21.74%), 11/44 (25.00%), and 11/44 (25.00%) in the high, medium, and low antibody-level groups, respectively. The one pig located between the predefined low- and medium-response thresholds was negative for PRRSV genome detection in both blood and throat swab samples. Individual-level post-immunization S/P values, antibody-response ranges, and PRRSV genome detection results are provided in Supplementary Table S2. As supportive clinical information, available individual-level reproductive records were reviewed and are provided in Supplementary Table S3. Abortion was recorded in 3 of the 135 pigs after the PRRSV infection experiment, corresponding to 2.22% of the infected subset and 7.50% of the 40 sows recorded as pregnant or pre-farrowing. These three abortion cases occurred at 5–7 days post-infection. At 15 days post-infection, all three pigs with abortion records were negative for PRRSV genome detection in both throat swabs and blood. Because respiratory signs such as cough were not systematically scored at the individual level, the percentage of pigs showing cough could not be reliably calculated. Serum ELISA analysis of the same 135 pigs showed that the numbers of pigs with S/P values of ≤0.9, 1.3–1.8, and ≥2.0 were 14, 24, and 67, respectively, accounting for 10.37%, 17.78%, and 49.63% of the infected subset (Figure 1C,E).

### 3.2. Analysis of Population Genetic Structure

**Figure 2:**
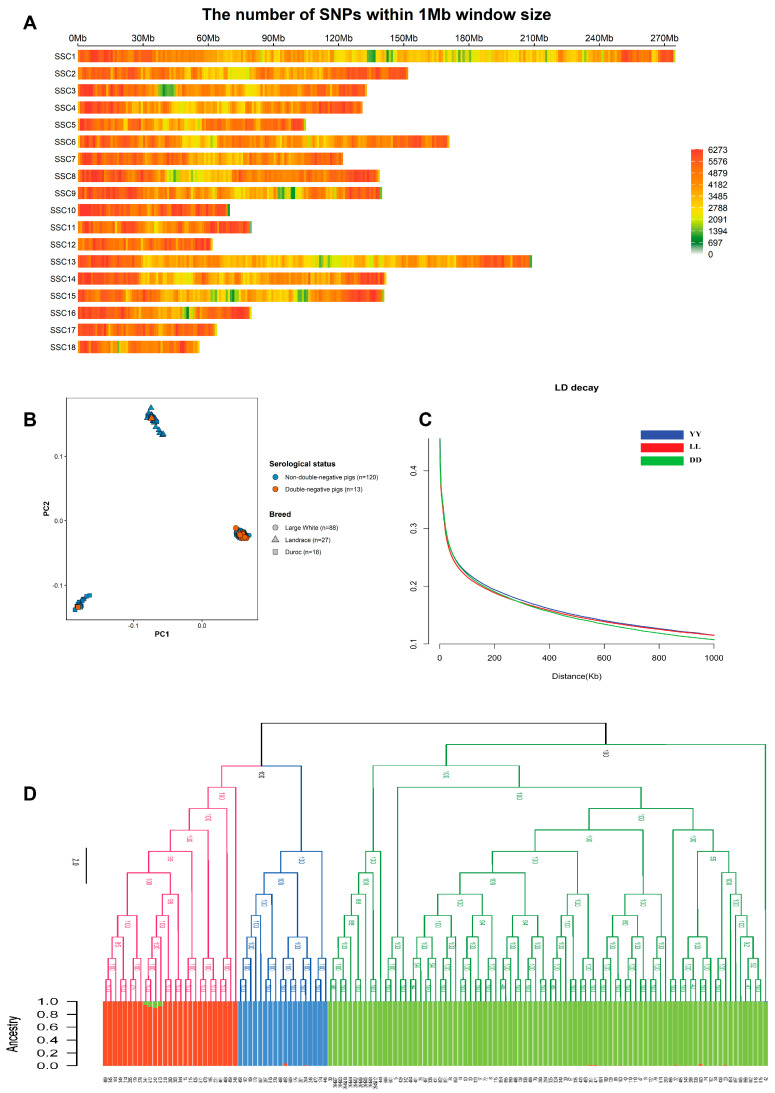
Overview of population genetic structure. (**A**) SNP density across the 18 autosomes calculated using 1-Mb windows. Chromosomes are labeled according to the standard Sus scrofa nomenclature as SSC1–SSC18. (**B**) Principal component analysis diagram. Double-negative pigs refer to individuals with a low antibody response (S/P ≤ 0.9) after immunization and infection, and with negative PRRSV detection in whole blood and throat swab samples post-infection; while non-double-negative pigs refer to the remaining sequenced pigs that do not simultaneously meet these two criteria. (**C**) Linkage imbalance attenuation diagram. (**D**) Pig herd structure at K = 3.

After quality control, the retained SNPs were broadly distributed across the pig genome, providing a suitable basis for subsequent population genetic analyses (Figure 2A). Principal component analysis (PCA) was annotated according to both the double-negative classification and breed. The PCA plot showed clear breed-related clustering, with Large White, Landrace, and Duroc pigs forming distinct clusters. Double-negative pigs were distributed within the corresponding breed clusters, and no separate cluster composed exclusively of double-negative pigs was observed (Figure 2B). Consistent with the PCA results, the maximum-likelihood phylogenetic tree showed that the 133 pigs clustered mainly into three population-related branches. ADMIXTURE analysis further supported this population structure, with the samples separated into three major ancestral components when K = 3 (Figure 2D).

Genome-wide linkage disequilibrium (LD) decay analysis showed that LD decreased rapidly with increasing physical distance. When r^2^ declined to 0.25, the corresponding genomic distance was approximately 50 kb (Figure 2C). Therefore, a 50-kb window size was used for subsequent window-based population genetic analyses. Together, these results characterized the genetic background and population structure of the experimental pigs, providing a population-level context for subsequent selection-signal and association analyses.

### 3.3. Genetic Diversity Index and Selection-Signal Detection

**Figure 3:**
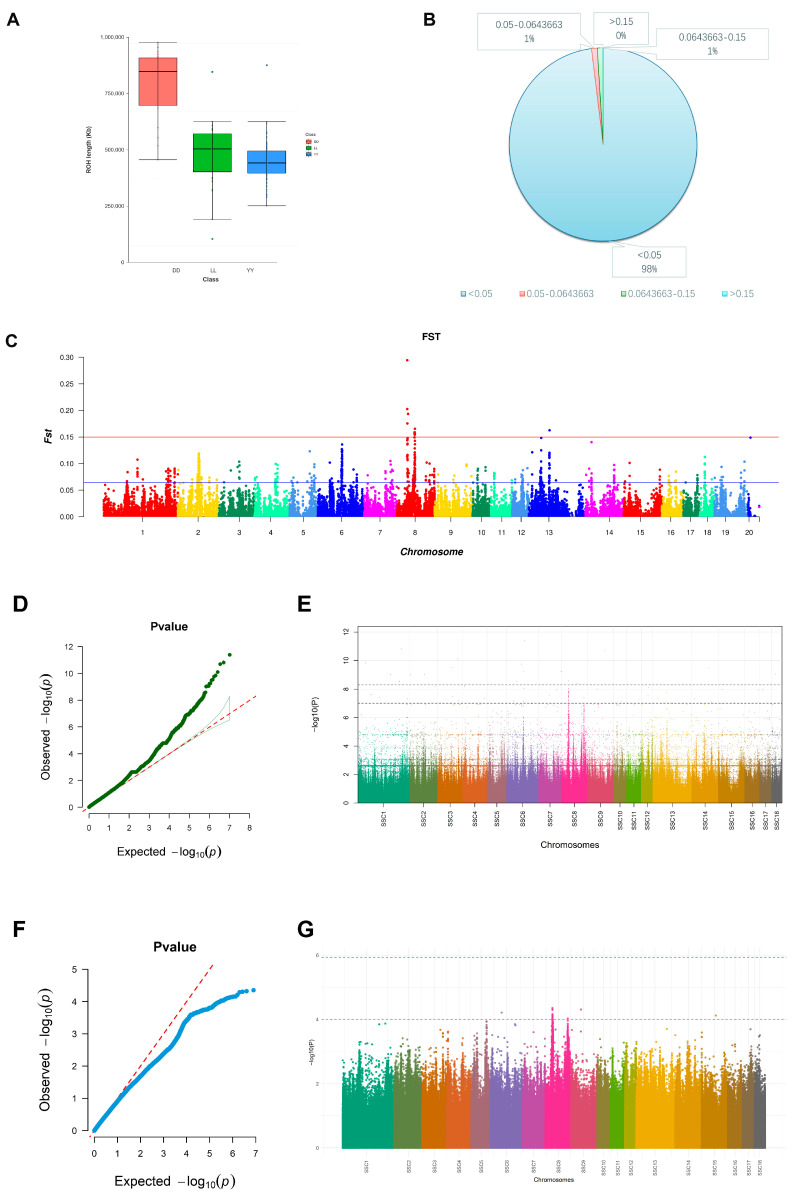
Genetic diversity, selection-signal detection, and genome-wide association analysis. (**A**) ROH analysis results. (**B**) Characteristic distribution of Fst values. (**C**) Manhattan diagram for Fst analysis of selected signals (Note: The red line is the Fst value > 0.15 threshold line, and the blue line is the Fst value > 0.0643663 threshold line). (**D**) Q-Q plot of the original unadjusted exploratory GWAS. (**E**) Manhattan plot of the original unadjusted exploratory GWAS. Only autosomal SNPs on SSC1–SSC18 are displayed, while the original threshold settings were retained. (**F**) Q-Q plot of the GWAS sensitivity analysis with PC1 and PC2 included as covariates. (**G**) Manhattan plot of the PC1+PC2-adjusted GWAS sensitivity analysis on SSC1–SSC18. In panel (**E**), the black dashed line indicates the original suggestive threshold calculated as 1/N, and the grey dashed line indicates the original Bonferroni-corrected threshold calculated as 0.05/N, where N = 10,245,129 represents the total number of SNPs used in the original GWAS threshold setting. In panel (**G**), the grey dashed line indicates an exploratory cutoff of P = 1 × 10^−4^, and the blue dashed line indicates the genome-wide suggestive threshold calculated as 1/Neff, where Neff = 852,896 represents the effective number of independent autosomal SNPs estimated after LD pruning.

Runs of homozygosity (ROH) analysis showed clear differences in homozygous fragment length among the three pig populations (Figure 3A). The Duroc population (DD) showed the highest ROH distribution, with a median of approximately 850 kb, indicating a relatively higher level of genomic homozygosity. The Landrace population (LL) showed an intermediate ROH distribution, with a median of approximately 500 kb. In contrast, the Large White population (YY) showed the lowest ROH distribution, with a median of approximately 450 kb, suggesting comparatively lower recent genomic homozygosity than the DD and LL populations.

Genome-wide Fst analysis was performed using 50 kb sliding windows to identify highly differentiated genomic regions between resistant and susceptible groups. Most windows showed low differentiation, with 47,056 windows having Fst values < 0.05. A total of 470 and 467 windows had Fst values of 0.05–0.0643663 and 0.0643663–0.15, respectively, whereas only 12 windows had Fst values > 0.15 (Figure 3B).

The top 1% Fst value (Fst > 0.0643663) was used as the suggestive threshold, and Fst > 0.15 was used as the high-differentiation threshold. In the Fst Manhattan plot, differentiated regions were mainly observed on chromosomes 8 and 13, with the strongest peak located on chromosome 8 (Figure 3C). The 12 windows with Fst values > 0.15 were annotated to several candidate genes, including *NFXL1*, *NIPAL1*, *CHIC2*, *LOC100623351*, *LOC100513671*, *LOC100513484*, *CENPC*, *STAP1*, *UBA6*, *GNRHR*, and *LOC100512727* (Table 2).

### 3.4. Genome-Wide Association Study

The original unadjusted exploratory GWAS was used as a discovery-stage scan to identify candidate association signals (Figure 3D,E). The GWAS cohort included 133 resequenced pigs, comprising 13 phenotype-coded cases and 120 controls as defined by the post-immunization and post-infection phenotypic screening criteria. In this scan, multiple top-ranked SNPs were detected and annotated to candidate genes including *PYGM*, *UIMC1*/*RAP80*, *SPAST*/*SPG4*, *NTF4*, *NFXL1*, *KIAA1324L*, and *FLNC* (Table 3). Considering the breed-related genetic structure observed in the PCA, GWAS was further performed with PC1 and PC2 included as covariates as a complementary PC-adjusted analysis (Figure 3F,G). The PC-adjusted GWAS showed no obvious genomic inflation $\left(\lambda_{GC}=0.933\right)$, although the overall *p*-value distribution became more conservative. Accordingly, GWAS signals were interpreted as exploratory discovery-stage signals and were further evaluated together with Fst, transcriptomic evidence, allele-frequency patterns, gene annotation, and previous biological evidence.

Candidate genes annotated from the original unadjusted GWAS were further compared with the results from the PC-adjusted GWAS (Table 3). Among these genes, *NFXL1* retained exploratory support after PC adjustment, with a top PC-adjusted SNP located in the *NFXL1* candidate region at SSC8:37,861,809 $\left(p=4.777\times 10^{-5}\right)$. Additional PC-adjusted exploratory signals were annotated to *TXK*, *LOC100524098*, *LOC110261044*, *ABCB11*, *CNGA1*, *FRYL*, *CORIN*, *NIPAL1*, and *EMCN* (Table 3).

### 3.5. Public Transcriptomic Analysis of Candidate Genes

**Figure 4:**
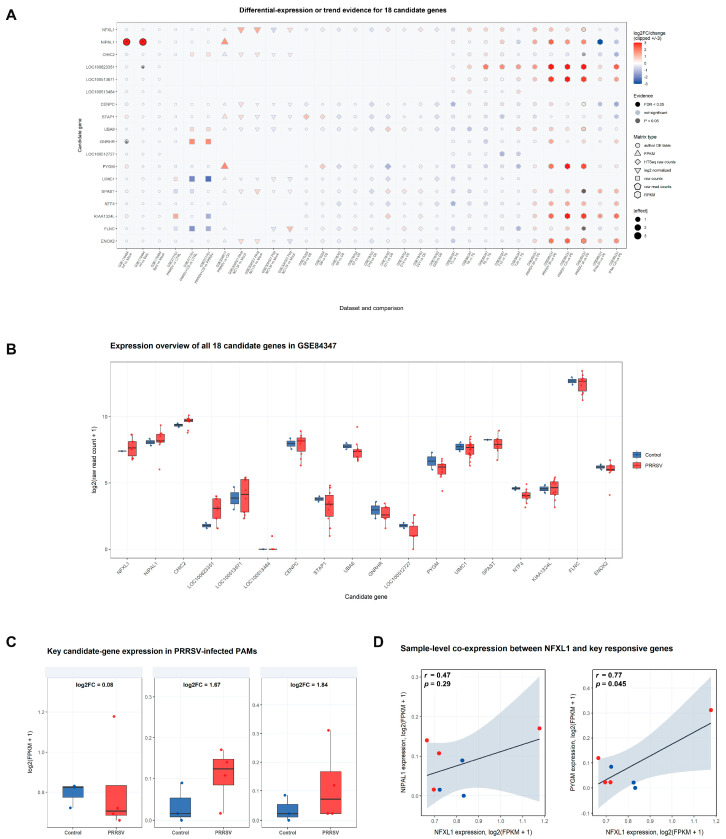
Public transcriptomic evidence supporting PRRSV candidate-gene prioritization. (**A**) Differential-expression and trend evidence for 18 candidate genes across public PRRSV-related transcriptomic datasets. (**B**) Expression overview of the 18 candidate genes in a representative PRRSV-related dataset. (**C**) Focused expression analysis of *NFXL1*, *NIPAL1*, and *PYGM* in PRRSV-infected porcine alveolar macrophages. (**D**) *NFXL1*-centered co-expression analysis with PRRSV-responsive candidate genes. In panels B–D, blue and red boxes/points indicate the control and PRRSV-infected groups, respectively. In panel D, the light-blue shaded areas represent the 95% confidence intervals of the fitted regression lines.

To provide expression-level context for candidate genes prioritized by selection-signal and GWAS analyses, the expression patterns of 18 candidate genes were examined across public PRRSV-related transcriptomic datasets. The integrated differential-expression and expression-trend summary showed that several candidate genes exhibited detectable expression changes or trends in PRRSV-related datasets, although the direction and magnitude varied among datasets, sample types, and comparisons (Figure 4A).

In the representative dataset GSE84347, sample-level expression profiles of the 18 candidate genes were further visualized (Figure 4B). Most candidate genes showed measurable expression in both control and PRRSV-infected samples, with several genes displaying visible group differences. Focused analysis of *NFXL1*, *NIPAL1*, and *PYGM* in PRRSV-infected porcine alveolar macrophages showed that *NFXL1* had only a minor expression change between control and infected samples (log_2_FC = 0.08, PRRSV-infected versus control), whereas *NIPAL1* and *PYGM* showed higher expression in infected samples, with log_2_FC values of 1.67 and 1.84, respectively (PRRSV-infected versus control; Figure 4C).

Pearson correlation analysis showed a positive correlation between *NFXL1* and *NIPAL1* expression that was not statistically significant (r = 0.47, p = 0.29), whereas *NFXL1* and *PYGM* showed a stronger positive correlation (r = 0.77, p = 0.045) (Figure 4D). Together, these analyses indicate that several genomic candidate genes are transcriptionally detectable and, in some cases, responsive in PRRSV-related datasets, providing external expression-level context for interpreting candidate genes prioritized by Fst and GWAS analyses.

### 3.6. Functional Enrichment and Variant-Level Characterization of Candidate Genes

**Figure 5:**
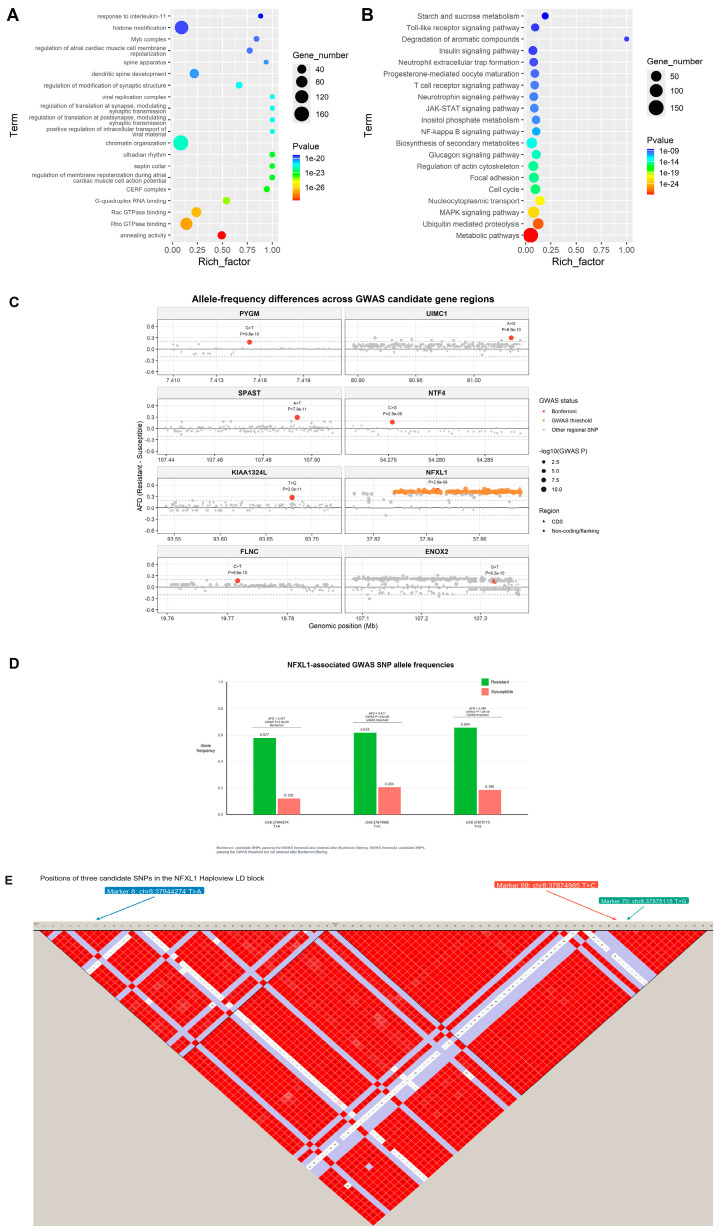
Functional enrichment of the integrated Fst and GWAS candidate gene set and variant-level evidence prioritizing the *NFXL1* candidate region. (**A**) GO enrichment analysis of the integrated Fst and GWAS candidate gene set. (**B**) KEGG enrichment analysis of the integrated Fst and GWAS candidate gene set. (**C**) Allele-frequency differences across annotated candidate-gene regions harboring Bonferroni-significant GWAS SNPs. (**D**) Allele-frequency comparison of *NFXL1*-associated GWAS SNPs between resistant and susceptible groups. (**E**) Linkage disequilibrium structure of the *NFXL1* candidate region on chromosome 8.

GO and KEGG enrichment analyses were performed using the integrated Fst and GWAS candidate gene set to describe the biological context of the prioritized candidates. GO enrichment showed that the integrated candidate genes were enriched in functional categories related to RNA binding, chromatin organization, GTPase binding, intracellular transport, and cellular regulatory processes (Figure 5A). KEGG enrichment further suggested associations with immune-related and signal-transduction pathways, including the Toll-like receptor, T cell receptor, Jak-STAT, MAPK, Fc gamma R-mediated phagocytosis, and ubiquitin-mediated proteolysis pathways (Figure 5B).

To assess variant-level patterns within candidate-gene regions, SNPs located in annotated candidate regions harboring Bonferroni-significant GWAS SNPs were extracted, and allele-frequency differences between resistant and susceptible groups were compared (Figure 5C). Positive values indicated higher allele frequencies in the resistant group, whereas negative values indicated higher allele frequencies in the susceptible group. Across the analyzed regions, the *NFXL1* region showed a cluster of SNPs with positive allele-frequency differences, while other candidate regions showed more localized or scattered patterns.

Three *NFXL1*-associated GWAS SNPs were further examined. The candidate alleles at chr8:37844274 T>A, chr8:37874985 T>C, and chr8:37875115 T>G showed higher frequencies in resistant pigs than in susceptible pigs, with frequencies of 0.577 versus 0.120, 0.615 versus 0.204, and 0.654 versus 0.185, respectively (Figure 5D). The haplotype-block heatmap of the *NFXL1* candidate region showed the positions of these three SNPs within the analyzed regional structure (Figure 5E). Haplotype-frequency analysis further showed that the A-C-G haplotype was more frequent in resistant pigs than in susceptible pigs (0.577 vs. 0.112), whereas the T-T-T haplotype was more frequent in susceptible pigs than in resistant pigs (0.806 vs. 0.346). Both haplotypes remained significant after both BH-FDR and Bonferroni correction, whereas the remaining low-frequency haplotypes did not remain significant after multiple-testing correction (Table 4). Together, these results provided functional context for the integrated Fst and GWAS candidate gene set and variant-level evidence supporting the prioritization of the *NFXL1* candidate region.

## 4. Discussion

Improving host genetic resistance provides a complementary strategy for PRRS control and disease-resistant pig breeding. Previous studies have shown that host genetic background contributes to variation in PRRSV response traits [5,7,8]. In the present study, resistant and susceptible groups were constructed using a two-stage phenotypic screening strategy that combined post-immunization antibody responses with post-infection PRRSV genome detection results and antibody profiles. Because post-immunization antibody response was included in the phenotypic screening strategy, the candidate genetic differences identified in this study may also be related to differences in antibody responses after modified-live PRRSV vaccination. However, ELISA S/P values alone cannot fully represent live-vaccine effectiveness. Therefore, whether these candidate variants influence the protective effect of live PRRSV vaccination should be further evaluated in future studies comparing pigs with different genotypes. This strategy was intended to distinguish pigs with divergent PRRS resistance-related phenotypic profiles by incorporating multiple post-immunization and post-infection indicators rather than relying on a single phenotypic measure. However, the strict screening process also reduced the final number of pigs retained for whole-genome resequencing, which should be considered when interpreting the statistical power of downstream analyses.

Population genetic analyses provided an important background for interpreting the subsequent selection-signal and association results. PCA, phylogenetic-tree analysis, and ADMIXTURE consistently separated the 133 pigs into three population-related groups, reflecting the genetic backgrounds of the commercial breeds included in this study. Genome-wide LD decay showed that r^2^ decreased to approximately 0.25 at around 50 kb, supporting the use of 50-kb sliding windows in subsequent window-based analyses. ROH analysis further revealed breed-related differences in genomic homozygosity, with Duroc showing the highest ROH level, Large White the lowest, and Landrace an intermediate pattern. Together, these results indicate that the study population had clear breed-related genetic structure and distinct homozygosity backgrounds, which should be considered when interpreting the Fst and GWAS results. This consideration is also relevant when comparing our results with previously reported breed-differentiation loci. Muñoz et al. analyzed genetic variability across major and candidate genes in European local pig breeds, including genes related to breed differentiation and production-related traits, such as *MC4R*, *TYRP1*, *IGF2*, *FUT1*, *RYR1*, *CAPNS1*, *KIT*, *AHR*, and *LEP* [1]. Some of these genes, such as *KIT* on SSC8, *FUT1*/*RYR1*/*CAPNS1* on SSC6, and *AHR* on SSC9, are located on chromosomes that also contained Fst or PC1+PC2-adjusted GWAS signals in the present study. However, this comparison indicates only chromosome-level overlap, rather than direct overlap with the candidate loci identified here. Therefore, these breed-differentiation loci should not be interpreted as evidence of shared PRRSV-response mechanisms. Instead, they highlight the need to consider breed-related genetic background when interpreting Fst and GWAS results in a multi-breed commercial population.

The integration of genome-wide selection-signal analysis and GWAS provided complementary evidence for prioritizing candidate loci associated with PRRS resistance-related phenotypes. Selection-signal analysis identified 11 candidate genes and revealed several highly differentiated regions on chromosome 8, among which the 37.85–37.90 Mb interval showed the strongest genome-wide differentiation signal, with an Fst value of 0.294582. In parallel, the original exploratory GWAS identified discovery-stage candidate association signals annotated to *PYGM*, *UIMC1*/*RAP80*, *SPAST*/*SPG4*, *NTF4*, *NFXL1*, *KIAA1324L*, and *FLNC*; after PC1/PC2 adjustment, *NFXL1* retained exploratory support, and additional PC-adjusted exploratory signals were observed. Considering the breed-related genetic structure observed in the PCA, the PC1/PC2-adjusted GWAS was interpreted as a complementary population-structure-adjusted analysis rather than as a replacement for the original exploratory scan. These results suggest that PRRS resistance-related phenotypes may be influenced by multiple genomic loci, although the identified signals should be interpreted as candidate evidence rather than direct proof of causal resistance genes.

Among these candidates, the chromosome 8 region containing *NFXL1* and *NIPAL1* is particularly noteworthy. *NFXL1* was supported by selection-signal evidence and retained exploratory support after PC adjustment, making it one of the most strongly prioritized genes in this study. *NFXL1* has been predicted to encode a transcription factor-like protein based on domain similarity, although its biological function remains poorly characterized [34]. Coding variants in *NFXL1* have also been associated with specific language impairment in humans, suggesting that genetic variation in this gene may have biological relevance, although this evidence is not directly related to PRRSV infection [35]. Given that host transcription factors can participate in PRRSV infection by regulating viral replication, inflammatory responses, and antiviral signaling processes [36], NFXL1 was retained as a genetically prioritized candidate for PRRS resistance-related phenotypes, rather than as a functionally validated PRRS-resistance gene. *NIPAL1*, located in the same chromosome 8 candidate region, may represent another biologically plausible candidate. It encodes a NIPA-like domain-containing protein and has been described as a magnesium transporter involved in magnesium-dependent cellular processes [37]. Although there is currently no direct evidence linking *NIPAL1* to PRRSV infection or PRRS resistance, its location within the highly differentiated chromosome 8 region and the presence of coding variation suggest that the chromosome 8 signal may reflect a regional candidate effect rather than a single-gene signal. *NFXL1* and *NIPAL1* may be involved in PRRSV resistance through different host-response mechanisms. *NFXL1* is predicted to be a transcription-regulatory factor, although its immune-related function remains poorly characterized [34]. Because PRRSV can antagonize innate immune signaling, including IRF3-, NF-κB-, and type I interferon-related pathways [38], variants linked to the NFXL1 candidate haplotype could potentially influence early antiviral transcriptional responses, including the regulation of interferon-stimulated or other innate immune genes. NIPAL1 provides another possible mechanism. As NIPAL1 encodes a magnesium influx transporter [37], its variants may affect intracellular magnesium homeostasis. Since PRRSV NSP9 functions as the viral RNA-dependent RNA polymerase [39], altered magnesium availability could theoretically influence cellular conditions related to viral RNA synthesis. However, these mechanisms remain hypothetical and require further validation by haplotype-stratified expression analysis, *NFXL1/NIPAL1* functional assays, intracellular magnesium measurement, and PRRSV replication assays. Several GWAS-annotated genes outside this region may also provide indirect functional clues. For example, *UIMC1*/*RAP80* participates in ubiquitin-mediated DNA damage-response processes and *BRCA1* recruitment at DNA damage sites [40,41], while ubiquitination is broadly involved in antiviral innate immune signaling, including RIG-I- and MDA5-mediated RNA virus sensing [42]. Nevertheless, there is currently no direct evidence linking these genes to PRRSV infection or PRRS resistance. Overall, the combined Fst and GWAS results prioritized several candidate regions and genes for further study, with the chromosome 8 region containing *NFXL1* and *NIPAL1* representing the most promising signal in this study. These findings should be interpreted in relation to previously studied PRRSV-response and resistance-related targets. The SSC4 region tagged by the WUR10000125 SNP, located near the *GBP* gene cluster, is a well-characterized naturally segregating host-response QTL and has been associated with viral load and weight gain following experimental PRRSV infection [5]. Within this QTL, a splice-site variant in GBP5 has been proposed as a candidate quantitative trait nucleotide [6]. In the present study, the strongest prioritized region was located on SSC8, especially around *NFXL1* and *NIPAL1*, and therefore did not directly overlap with the previously reported SSC4 WUR/*GBP5* QTL. This difference should not be interpreted as evidence against the established WUR/*GBP5* effect but may reflect differences in phenotype definition, population composition, sample size, PRRSV strain or infection model, and analytical strategy. In contrast, *CD163*-based gene-editing studies target a PRRSV entry receptor and have shown that loss or domain-specific modification of *CD163*, particularly the SRCR5 domain, can confer strong resistance to PRRSV infection [9,10]. Compared with these established targets, the chromosome 8 *NFXL1*/*NIPAL1* region identified here should be interpreted as a novel candidate host-response region, rather than as a validated resistance locus or a replacement for WUR/*GBP5* or *CD163*. This signal was prioritized using a different phenotype-guided strategy based on post-immunization antibody response, post-infection PRRSV genome detection, population differentiation, GWAS, transcriptomic evidence, and haplotype analysis. Further validation in independent populations and functional assays will be required to confirm the roles of *NFXL1* and *NIPAL1* in PRRSV-related host response.

Public transcriptomic analysis provided independent expression-level evidence for interpreting the candidate genes identified by selection-signal analysis and GWAS. Because genomic signals alone cannot determine whether candidate genes are expressed or transcriptionally regulated under PRRSV-related conditions, integrating public transcriptomic datasets helped place these candidates into infection-relevant biological contexts. The observation that several candidate genes were detectable or showed expression changes across PRRSV-related datasets suggests that part of the genomic candidate set is also represented at the transcriptomic level in immune cells or tissues relevant to PRRSV infection. However, these transcriptomic results should be interpreted as supportive evidence rather than direct functional validation of the candidate genes.

Among the focused candidate genes, *NIPAL1* and *PYGM* showed increased expression in PRRSV-infected porcine alveolar macrophages, suggesting that these genes may be transcriptionally responsive to PRRSV infection in macrophages. This finding is relevant because porcine alveolar macrophages are major target cells for PRRSV infection [43], and genes showing infection-associated expression changes in these cells may provide useful expression-level context for interpreting genomic candidates. In contrast, *NFXL1* showed only a minor expression change in the same dataset. Therefore, *NFXL1* should be interpreted primarily as a genetically prioritized candidate gene rather than as a transcriptome-supported differentially expressed gene. Importantly, the limited differential-expression signal of *NFXL1* in the examined public transcriptomic datasets does not exclude its potential relevance, because genetic variants may affect gene function, regulatory activity, or downstream pathways without necessarily causing marked changes in transcript abundance. Differences in sample type, infection stage, experimental design, and sample size may also influence the detectability of expression changes.

Functional enrichment analysis indicated that the prioritized candidate gene set was enriched in several pathways relevant to PRRSV–host interactions. The enrichment of Toll-like receptor and MAPK signaling pathways is consistent with previous evidence that PRRSV infection can activate inflammatory signaling in porcine alveolar macrophages, including downstream NF-kappa B-related responses. For example, PRRSV has been reported to induce IL-1β production through the TLR4/MyD88 pathway and downstream NF-kappa B, ERK1/2, and p38 MAPK signaling [44]. PRRSV infection can also activate p38 and JNK pathways, and inhibition of these pathways reduces viral RNA synthesis, viral protein expression, progeny virus production, and virus-induced cytokine responses [36]. The enrichment of Jak-STAT signaling and ubiquitin-mediated proteolysis further supports the relevance of antiviral immune regulation. PRRSV nsp1β can antagonize interferon-activated JAK/STAT signaling by promoting karyopherin-α1 degradation and inhibiting STAT1 nuclear translocation [45], while PRRSV nucleocapsid protein can suppress RIG-I-mediated antiviral signaling by interfering with *TRIM25*-mediated RIG-I ubiquitination [46]. Therefore, the enrichment of inflammatory, interferon-related, and ubiquitin-dependent pathways supports the biological relevance of the integrated candidate gene set in PRRSV-related host responses, although these pathway-level findings should be interpreted as supportive evidence rather than direct functional validation of any single candidate gene.

Variant-level analysis provided additional support for interpreting the *NFXL1* candidate region. Compared with single-gene annotation alone, the combination of allele-frequency comparison, LD analysis, and haplotype-frequency analysis offered a more regional view of the genetic background surrounding *NFXL1*. For the three *NFXL1*-associated candidate SNPs, the alleles chr8:37844274-A, chr8:37874985-C, and chr8:37875115-G showed higher frequencies in resistant pigs than in susceptible pigs, indicating a distinct allele-frequency pattern in this region. Haplotype analysis further showed that, after multiple-testing correction across the five tested haplotypes, A-C-G was more frequent in resistant pigs, whereas T-T-T was more frequent in susceptible pigs. This haplotype-level difference suggests that the *NFXL1* region may harbor genetic variation associated with PRRS resistance-related phenotypic differences.

Although the two-stage phenotypic screening strategy incorporated multiple post-immunization and post-infection indicators, the strict screening criteria resulted in a relatively small GWAS cohort, which may limit statistical power and should be considered when interpreting the association results. Because the study was conducted in a commercial farm population with pigs at different productive stages, the potential influence of population heterogeneity should be considered, and further validation in independent populations is needed. Although the pigs were clinically healthy at the time of inclusion, comprehensive laboratory screening for other viral, bacterial, or parasitic pathogens was not performed. Therefore, potential subclinical co-infections or secondary infections could not be completely excluded, which may have introduced additional uncertainty into the interpretation of PRRSV-related phenotypes. In addition, the public transcriptomic datasets used in this study differed in sample type, infection stage, experimental design, and sample size and therefore should be interpreted as external expression-level evidence rather than functional validation. Future studies should expand validation in independent pig populations and further evaluate the candidate genes and variants through expression validation by qPCR, regional fine-mapping, and functional assays such as gene knockdown or overexpression followed by PRRSV infection. Overall, these findings provide useful genetic and biological clues for understanding PRRS resistance-related phenotypes and may help inform future marker-assisted selection and disease-resistance breeding. Taken together, these limitations indicate that the present findings should be regarded as association-based candidate evidence for PRRSV resistance-related phenotypic variation, rather than as direct evidence of biological causality.

## 5. Conclusions

In summary, this study aimed to better understand pig resistance to PRRSV by constructing groups with different PRRS resistance-related phenotypic profiles through post-immunization and post-infection phenotypic screening, followed by selection-signal analysis and GWAS. Candidate genes and SNPs were prioritized through integrated selection-signal analysis, exploratory GWAS, PC-adjusted analysis, transcriptomic comparison, and variant-level analyses. Public transcriptomic analysis provided additional expression-level evidence for selected candidate genes, especially *NIPAL1* and *PYGM*. Functional enrichment and variant-level analyses further supported the prioritized candidate gene set, particularly the *NFXL1* candidate region. In this region, the A-C-G haplotype was more frequent in resistant pigs, whereas the T-T-T haplotype was more frequent in susceptible pigs. Given the high genetic variability of PRRSV, breeding for a single resistance-associated haplotype may carry a potential risk of viral escape under strong selection pressure. Therefore, the candidate haplotype identified in this study should be regarded as a preliminary genetic clue rather than a single decisive breeding target. Future applications should combine independent population validation, evaluation against different PRRSV strains, multi-locus breeding strategies, and continuous viral surveillance. These candidate SNPs, genes, and haplotypes provide preliminary association-based genetic evidence for PRRSV resistance-related phenotypic variation in pigs, but they should not be interpreted as confirmed causal determinants of PRRS resistance. Further independent population validation and functional studies are needed to clarify their biological roles.

## Figures and Tables

**Table 1:** Statistics of PRRSV genome test results after PRRS infection.

Test Type	PRRSV Genome	Number of Individuals	Percentage
Throat Swab	Negative	107	79.26%
Throat Swab	Positive	28	20.74%
Blood	Negative	126	93.33%
Blood	Positive	9	6.67%

**Table 2:** Regions with Fst values greater than 0.15.

Chromosome	Position_Start	Position_End	*Fst*	Gene
8	37850001	37900000	0.294582	*NFXL1*
8	37900001	37950000	0.202608	*NIPAL1*
8	40800001	40850000	0.193382	*CHIC2*
8	38250001	38300000	0.175529	NA
8	65950001	66000000	0.165414	*LOC100623351*
13	76000001	76050000	0.162676	NA
8	65250001	65300000	0.159022	NA
8	64550001	64600000	0.158709	NA
8	65850001	65900000	0.156727	*LOC100513671* *LOC100513484*
8	65400001	65450000	0.155922	NA
8	65300001	65350000	0.155758	*CENPC* *STAP1*
8	65450001	65500000	0.154177	*UBA6* *GNRHR* *LOC100512727*

NA indicates that no annotated gene was identified within the corresponding window.

**Table 3:** Comparison of autosomal original exploratory GWAS signals with GWAS results adjusted for PC1 and PC2.

Gene	Original GWAS	Original *p*	PC-Adjusted GWAS	PC-Adjusted *p*	Evidence
*PYGM*	SSC2:7415271	9.647 × 10^−10^	NA	NA	Original only
*UIMC1 (RAP80)*	SSC2:81032176	8.922 × 10^−10^	NA	NA	Original only
*SPAST (SPG4)*	SSC3:107494014	7.90 × 10^−11^	NA	NA	Original only
*NTF4*	SSC6:54275421	2.86 × 10^−9^	NA	NA	Original only
*NFXL1*	SSC8:37844274	2.62 × 10^−9^	SSC8:37861809	4.777 × 10^−5^	Retained after PC adjustment
*KIAA1324L*	SSC9:93678594	1.99 × 10^−11^	NA	NA	Original only
*FLNC*	SSC18:19771716	8.63 × 10^−10^	NA	NA	Original only
*TXK*	NA	NA	SSC8:37979358	4.432 × 10^−5^	PC-adjusted signal
*LOC100524098*	NA	NA	SSC9:51712511	4.955 × 10^−5^	PC-adjusted signal
*LOC110261044*	NA	NA	SSC6:58554915	6.184 × 10^−5^	PC-adjusted signal
*ABCB11*	NA	NA	SSC15:75444864	7.588 × 10^−5^	PC-adjusted signal
*CNGA1*	NA	NA	SSC8:37914100	7.918 × 10^−5^	PC-adjusted signal
*FRYL*	NA	NA	SSC8:38709117	8.089 × 10^−5^	PC-adjusted signal
*CORIN*	NA	NA	SSC8:37788801	8.917 × 10^−5^	PC-adjusted signal
*NIPAL1*	NA	NA	SSC8:37962256	9.248 × 10^−5^	PC-adjusted signal
*EMCN*	NA	NA	SSC8:120112743	9.468 × 10^−5^	PC-adjusted signal

Note: The original GWAS was used as an exploratory discovery scan. The GWAS with PC1 and PC2 included as covariates was used as a sensitivity analysis to evaluate the potential influence of population structure. PC-adjusted signals were defined as *p* < 1 × 10^−4^ and were not considered genome-wide significant. NA indicates that no corresponding gene-level exploratory signal was identified in the indicated analysis. Original only, supported only by the original exploratory GWAS; PC-adjusted signal, identified only in the PC-adjusted sensitivity analysis.

**Table 4:** *NFXL1* haplotype frequencies in resistant and susceptible groups.

Haplotype	Resistant	Susceptible	Raw *p*	BH-FDR Adjusted *p*	Bonferroni Adjusted *p*	Significance After FDR
A-C-G	0.577	0.112	1.99 × 10^−7^	9.95 × 10^−7^	9.95 × 10^−7^	Significant
T-T-T	0.346	0.806	2.41 × 10^−6^	6.03 × 10^−6^	1.21 × 10^−5^	Significant
T-C-G	0.038	0.065	1.000	1.000	1.000	Not significant
T-T-G	0.038	0.000	0.101	0.168	0.505	Not significant
T-C-T	0.000	0.013	1.000	1.000	1.000	Not significant

## Data Availability

The whole-genome resequencing and phenotype data generated in this study are available from the corresponding authors upon reasonable request. Public transcriptomic datasets analyzed in this study are available from the NCBI Gene Expression Omnibus under accession numbers GSE174494, GSE89331, GSE296917, GSE277761, GSE304527, GSE84347, GSE75304, and GSE78762. Other data supporting the findings of this study are included in the article and its Supplementary Materials.

## Supplementary Materials

The following supporting information can be downloaded at: https://www.mdpi.com/article/10.3390/ani16142218/s1, Figure S1: Agarose gel electrophoresis results of DNA from some samples; Figure S2: Phylogenetic tree analysis diagram; Figure S3: Candidate gene co-expression analysis; Figure S4: GO Level 2 functional classification of genes in selection-signal regions; Figure S5: Top 20 enriched KEGG pathways of genes in selection-signal regions; Figure S6: KEGG Level 1 pathway classification of genes in selection-signal regions; Figure S7: GO Level 2 functional classification of significant genes from the genome-wide association study after Bonferroni correction; Figure S8: Top 20 enriched KEGG pathways of significant genes from the genome-wide association study after Bonferroni correction; Figure S9: KEGG Level 1 pathway classification of significant genes from the genome-wide association study after Bonferroni correction; Figure S10: Haplotype block analysis of chr2:7408271–7422271; Figure S11: Haplotype block analysis of chr3:107488014–107500014; Figure S12: Haplotype block analysis of chr8:37839274–37849274; Figure S13: Haplotype block analysis of chr9:93662594–93694594; Figure S14: Haplotype block analysis of chr18:19760716–19782716; Table S1: Metadata of the initial 699 pigs; Table S2: PRRSV genome detection by antibody-response group; Table S3: Abortion records after PRRSV infection; Table S4: Ct values of PRRSV-positive samples at 15 dpi; Table S5: Phenotype population selection and GWAS; Table S6: GO and KEGG enrichment; Table S7: Haplotype analysis; Table S8: Public transcriptomic validation.

## Abbreviations

The following abbreviations are used in this manuscript:

PRRS	Porcine reproductive and respiratory syndrome
PRRSV	Porcine reproductive and respiratory syndrome virus
SNP	Single-nucleotide polymorphism
GWAS	Genome-wide association study
Fst	Fixation index
LD	Linkage disequilibrium
ROH	Runs of homozygosity
ELISA	Enzyme-linked immunosorbent assay
PCR	Polymerase chain reaction
