# Supplementary material for: Whole-Genome Resequencing-Based Selection-Signal and Association Analyses Prioritize Candidate Genes and Haplotypes for PRRS Resistance-Related Traits in Pigs
Source: Animals (Basel). 2026 Jul 17;16(14):2218. doi: 10.3390/ani16142218 (PMC13406021; doi:10.3390/ani16142218)
Supplement: Supplementary file 1 [file animals-16-02218-s001.zip › Supplementary_Figures.pdf]

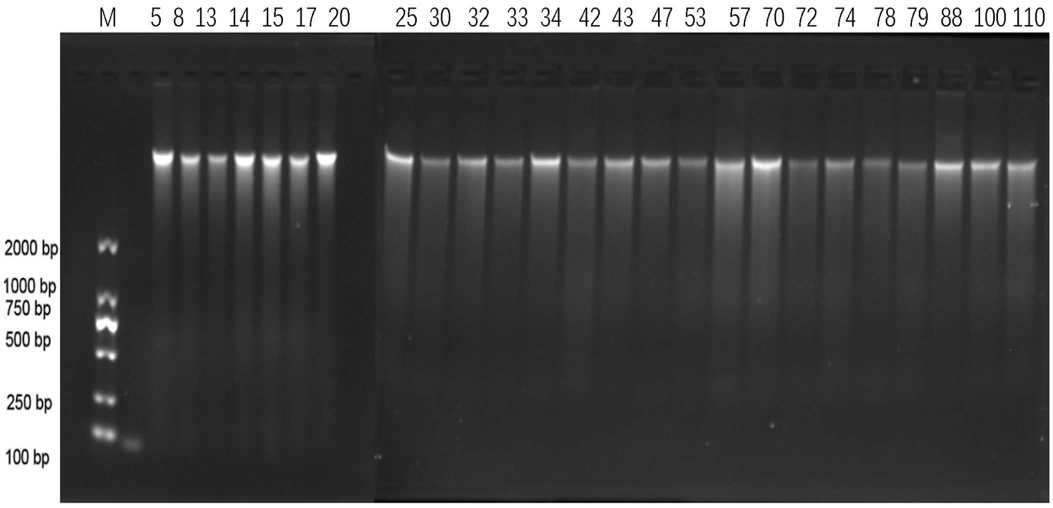

Figure S1: Agarose gel electrophoresis results of DNA from some samples.

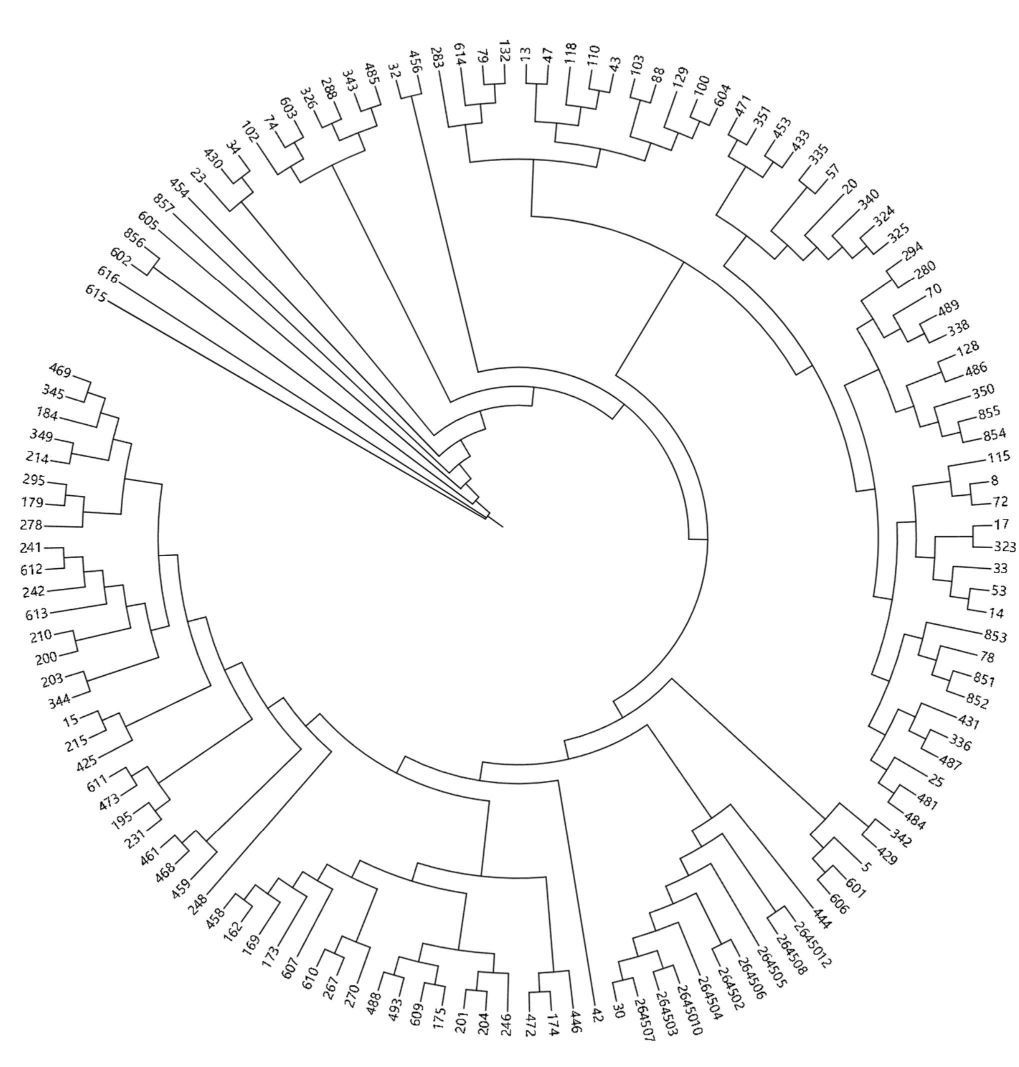

Figure S2: Phylogenetic tree analysis diagram.

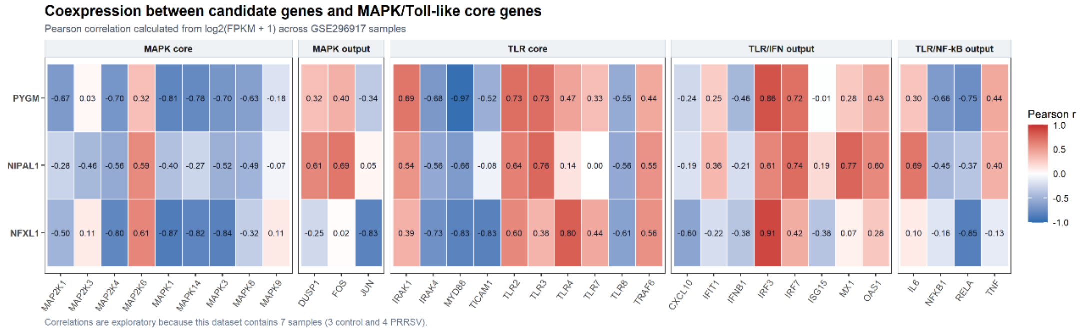

Figure S3: Candidate Gene Co-expression Analysis.

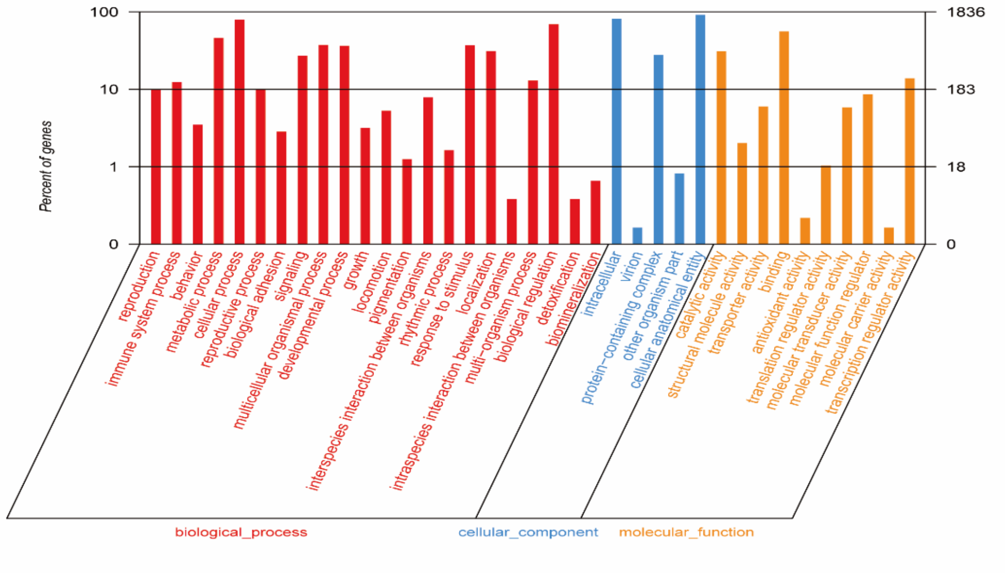

Figure S4: GO Level 2 functional classification of genes in selection-signal regions.

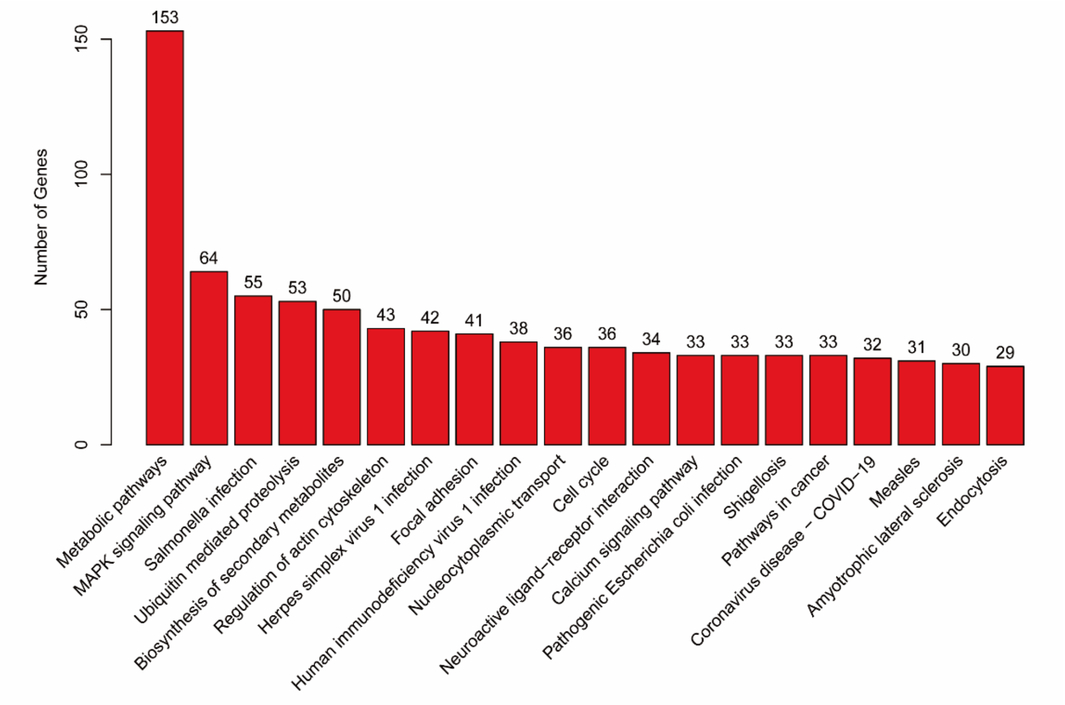

Figure S5: Top 20 enriched KEGG pathways of genes in selection-signal regions.

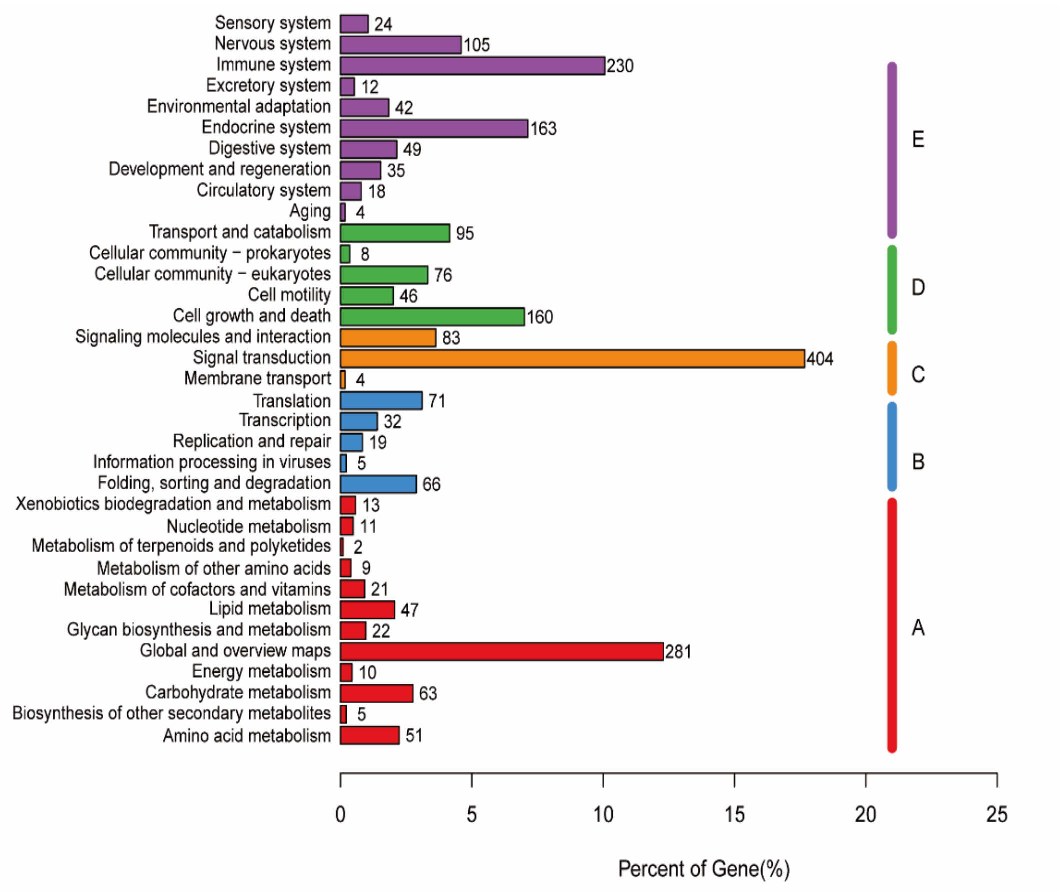

Figure S6: KEGG Level 1 pathway classification of genes in selection-signal regions.

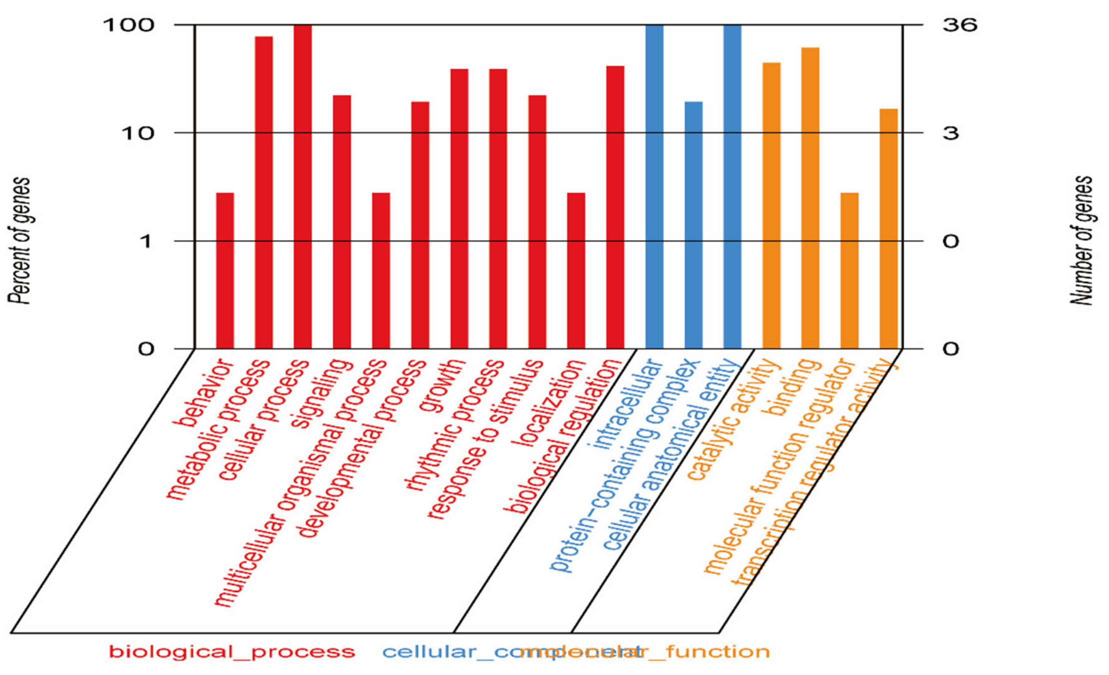

Figure S7: GO Level 2 functional classification of significant genes from the genome-wide association study after Bonferroni correction.

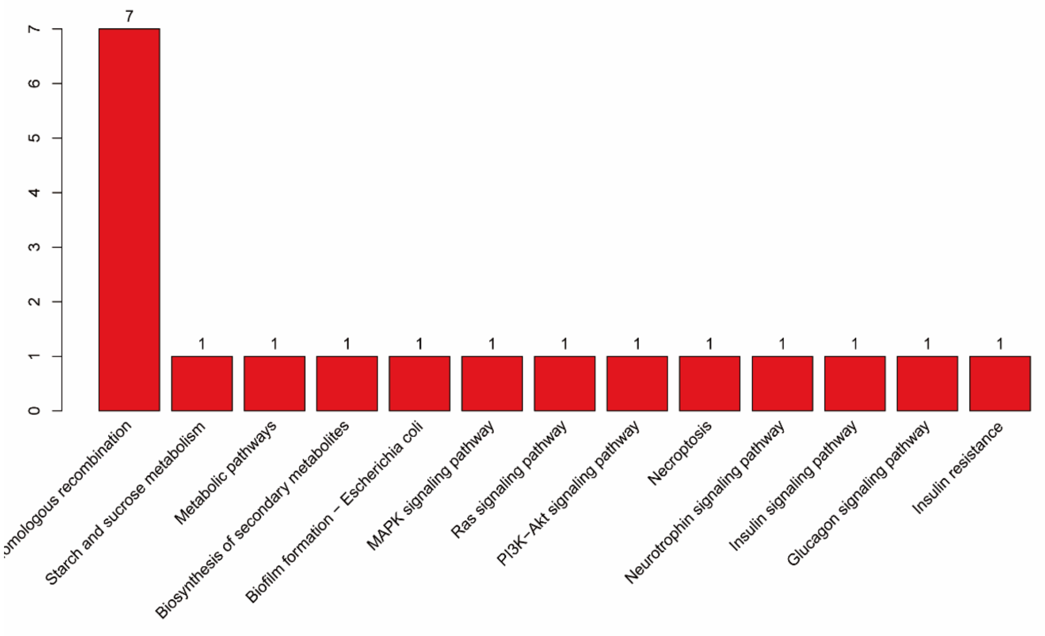

Figure S8: Top 20 enriched KEGG pathways of significant genes from the genome-wide association study after Bonferroni correction.

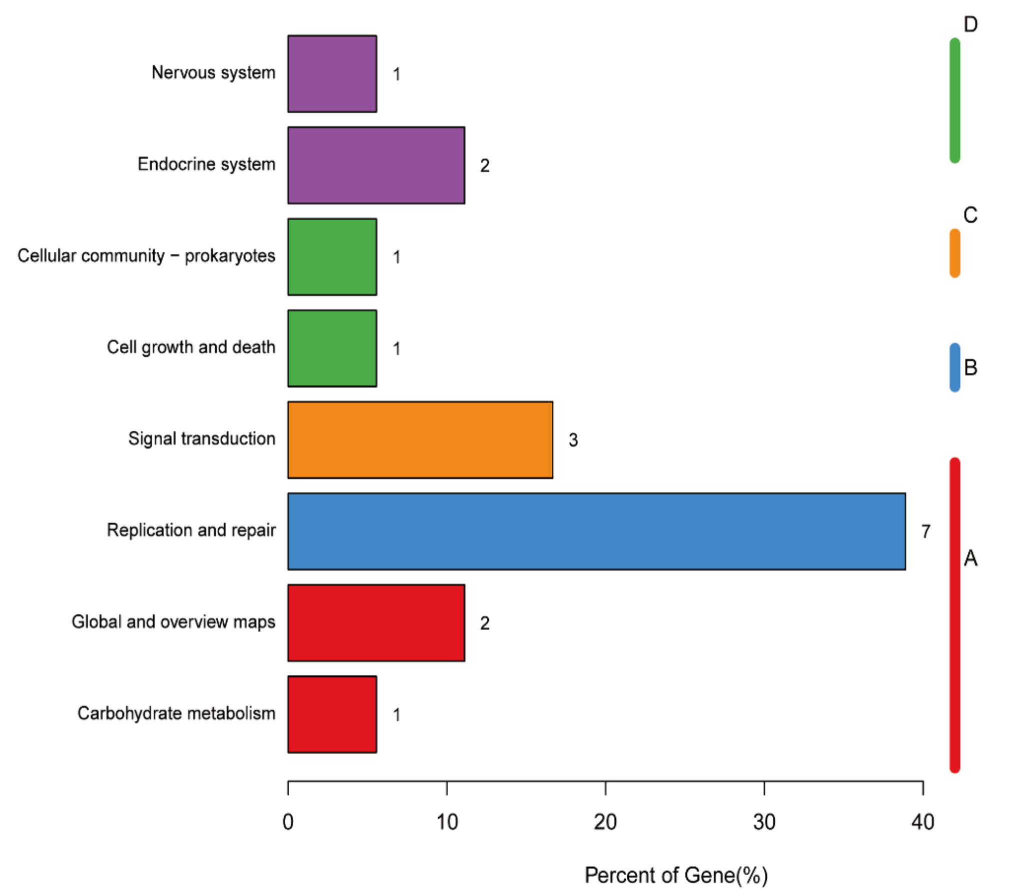

Figure S9: KEGG Level 1 pathway classification of significant genes from the genome-wide association study after Bonferroni correction.

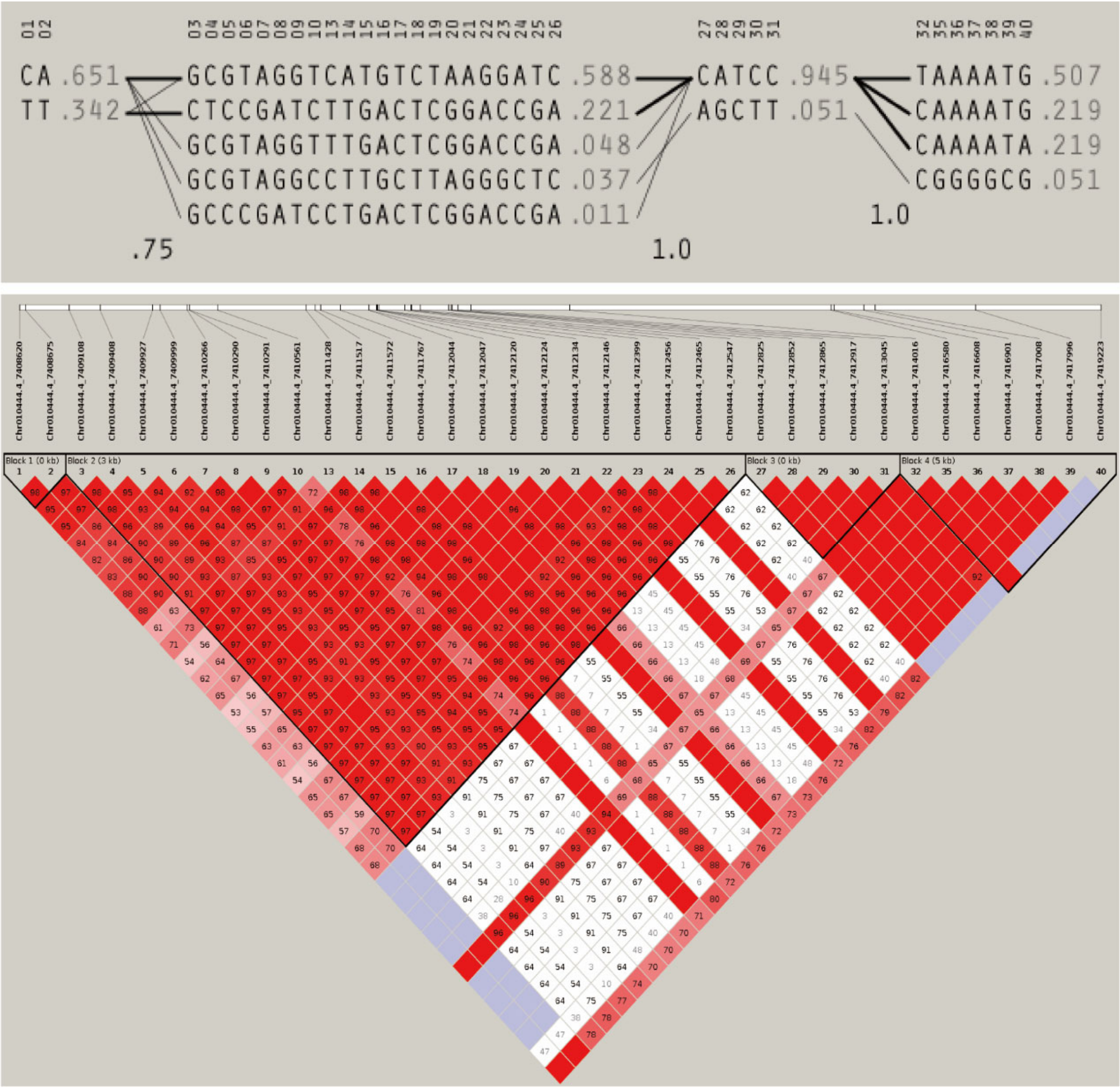

Figure S10: Haplotype block analysis of chr2:7408271-7422271.

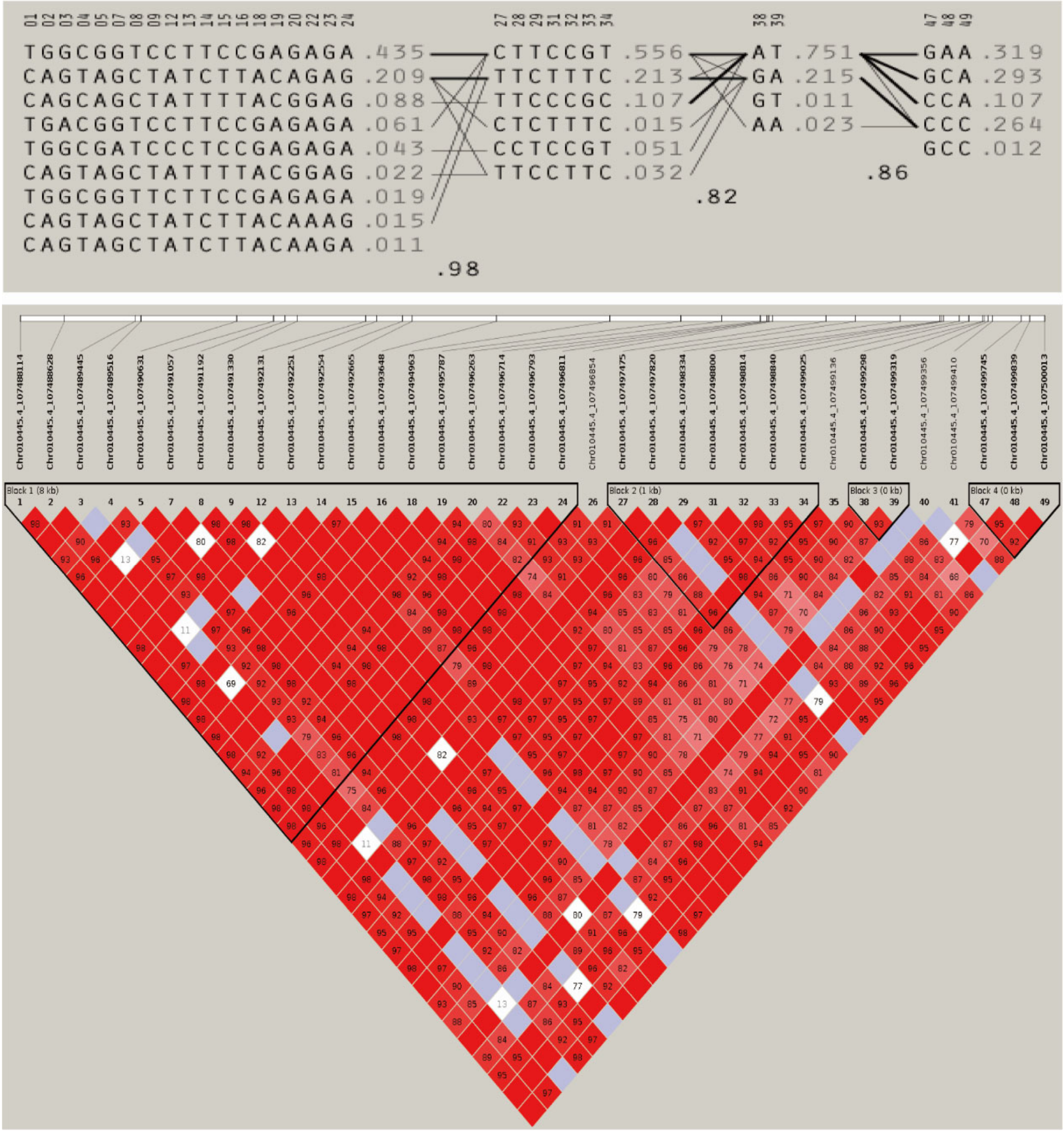

Figure S11: Haplotype block analysis of chr3:107488014-107500014.

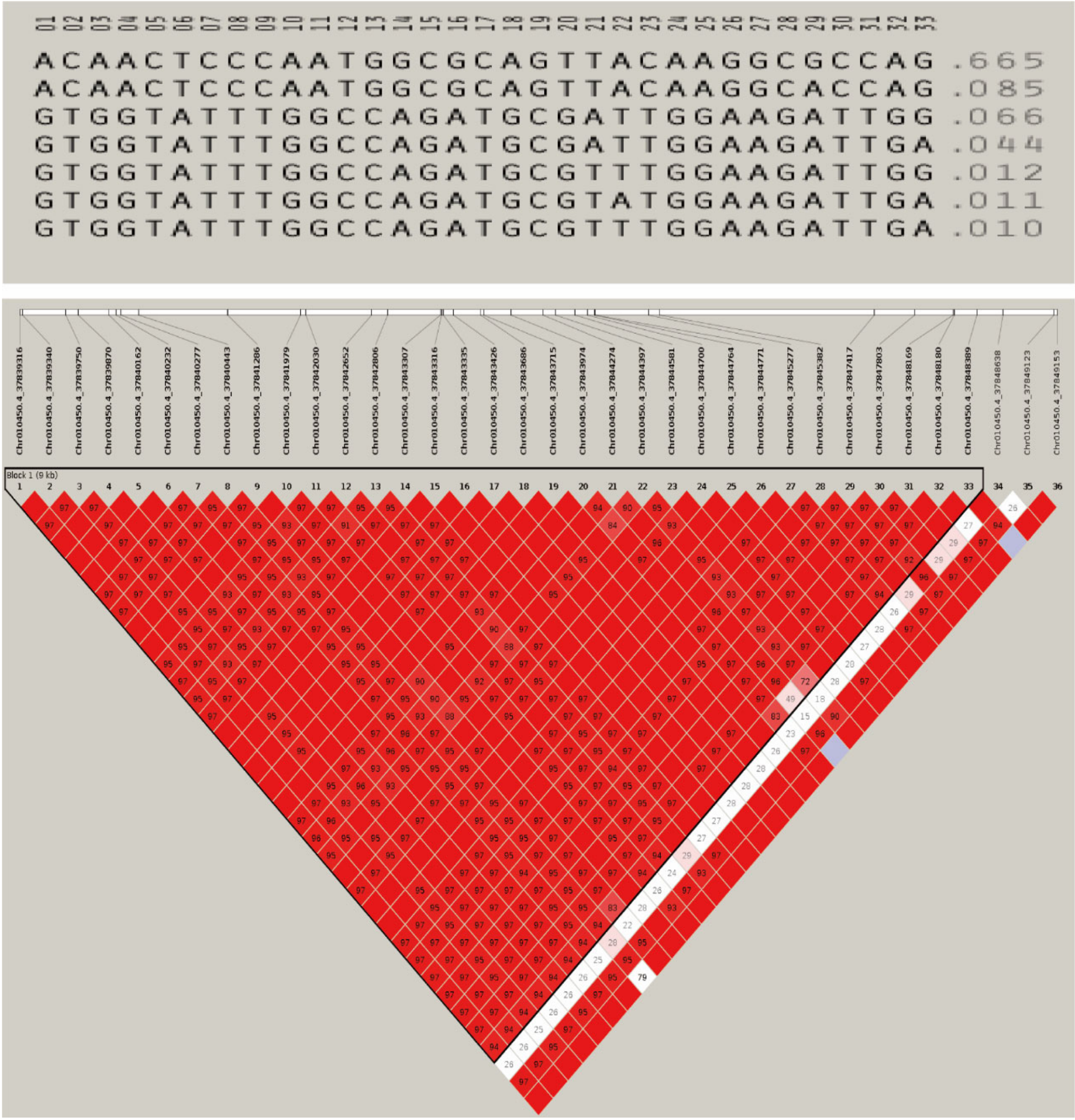

Figure S12: Haplotype block analysis of chr8:37839274-37849274.

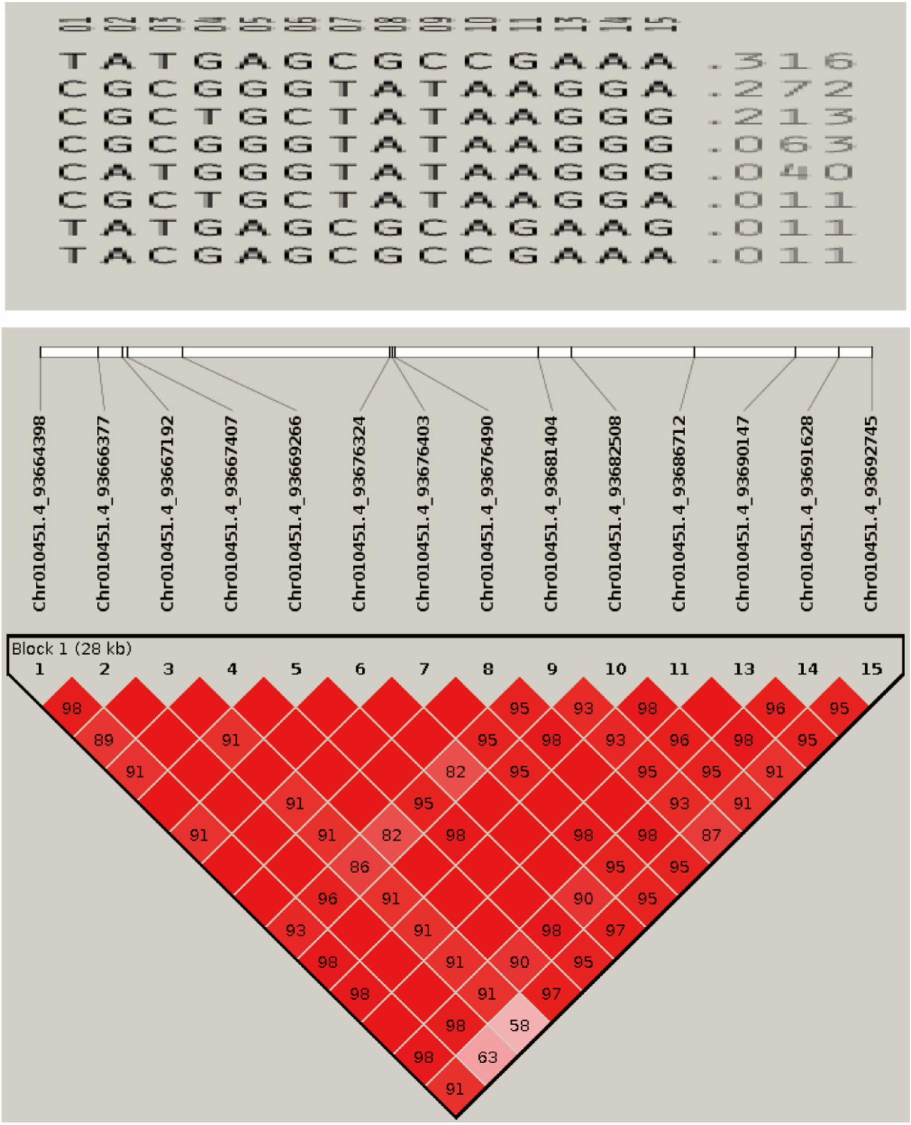

Figure S13: Haplotype block analysis of chr9:93662594-93694594.

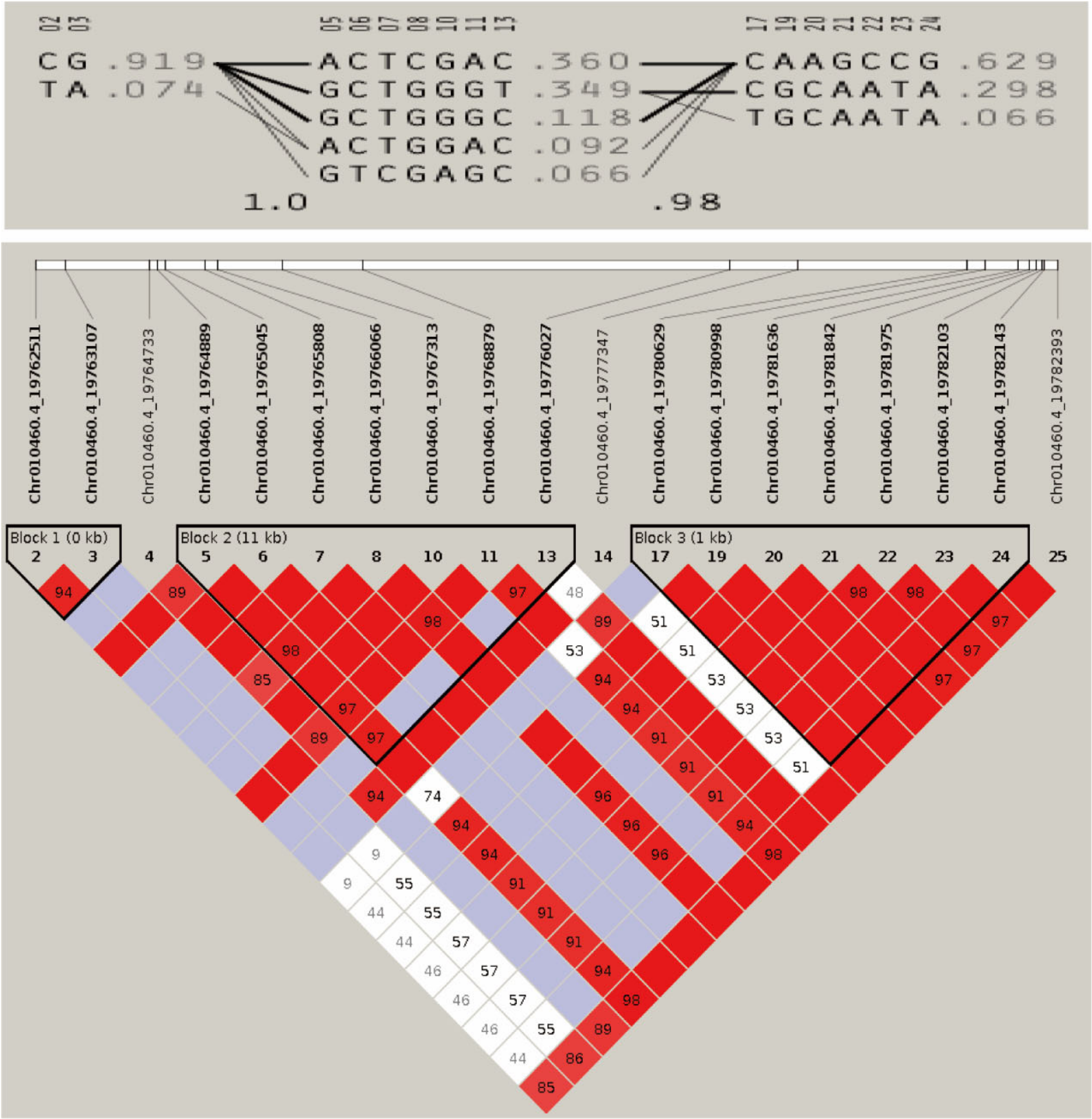

Figure S14: Haplotype block analysis of chr18:19760716-19782716.
